# Predicting Chronic Kidney Disease from Biomarkers: An Explainable Machine Learning Approach

**DOI:** 10.3390/diagnostics16132000

**Published:** 2026-06-26

**Authors:** Abass Al-Momany, Omar Almomani, Ensaf Y. Almomani

**Affiliations:** 1 Department of Medical Laboratory Sciences, The University of Jordan, Queen Rania St, Amman 11942, Jordan; a.momany@ju.edu.jo; 2 Department of Networks and Cybersecurity, Faculty of Information Technology, Al-Ahliyya Amman University, Amman 19328, Jordan; 3 Department of Basic Medical Sciences, Faculty of Medicine, Al-Balqa Applied University, Al-Salt 19117, Jordan; ensaf.momani@bau.edu.jo

**Keywords:** chronic kidney disease (CKD), biomarker-based prediction, explainable machine learning

## Abstract

**Background/Objectives**: Chronic kidney disease (CKD) remains underdiagnosed until advanced stages, motivating reliable, clinically deployable screening models that pair high discrimination with an explicit operating threshold and transparent explanations. **Methods**: In this study, we propose a CKD detection framework that integrates structured preprocessing, class imbalance handling, stratified 10-fold cross-validation with out-of-fold (OOF) prediction, and clinically oriented threshold selection via the Youden index, followed by explainability using SHAP and LIME. Experiments were conducted on two datasets. Across a broad panel of ten machine learning models, gradient boosting methods consistently dominated. **Results**: LightGBM achieved the best overall clinical composite performance on both datasets. On Dataset 1, LightGBM delivered near-ceiling OOF discrimination (ROC-AUC = 99.98, PR-AUC = 99.98) and an excellent clinically balanced performance at the best Youden threshold (0.41), reaching sensitivity = 99.20, specificity = 99.60, accuracy = 99.40, F1 = 99.40, and MCC = 98.80, with robust cross-validation stability (CV AUC = 99.99 ± 0.04; CV sensitivity = 99.10 ± 1.81; CV specificity = 99.46 ± 1.42; CV MCC = 98.59 ± 2.19), strong calibration (Brier = 0.006), and fast training (0.078 ± 0.019 s/fold). On Dataset 2, LightGBM maintained high generalization (ROC-AUC = 99.72, PR-AUC = 99.64) and clinically deployable balance at the best Youden threshold (0.35), achieving sensitivity = 98.10, specificity = 98.03, accuracy = 98.06, F1 = 98.06, and MCC = 96.13, with consistent fold-wise performance (CV AUC = 99.69 ± 0.25; CV sensitivity = 97.25 ± 1.25; CV specificity = 98.11 ± 1.02; CV MCC = 95.37 ± 1.56), acceptable calibration (Brier = 0.0173), and practical training time (0.742 ± 0.144 s/fold). **Conclusions**: Finally, SHAP and LIME explanations confirmed that model decisions align with clinically meaningful renal function and symptom/biomarker patterns at both population and patient levels, supporting safer translation of the proposed framework into CKD screening and decision-support workflows.

## 1. Introduction

Chronic kidney disease (CKD) is characterized by a permanent decline in glomerular filtration rate (GFR < 60 mL/min/1.73 m^2^ for ≥3 months) or signs of structural or functional damage in the renal structures [1,2]. Some of the top etiologies include diabetes, hypertension, and glomerulonephritis, all of which contribute to the significant burden of CKD in the world, high healthcare expenditures, and elevated morbidity and mortality risk [3,4]. Timely outcomes and early identification of the main risk factors are thus vital in maximizing the management of CKD and the outcomes of patients. Nonetheless, traditional clinical evaluation and laboratory analysis might not necessarily provide adequate, constant or personalized forecasting, especially in cases where biomarker patterns are not complete or in cases where numerous clinical variables interact in a nonlinear manner. As a result, there is an increasing need to develop computational methods that have the capability of incorporating common clinical biomarkers to aid with strong prediction and clinically significant risk stratification [5,6,7,8].

CKD is a major world health issue that affects more than 700 million people, which is about 10 percent of the global population [9]. Epidemiological studies also reveal that a high percentage of CKD patients are found in the low- and middle-income nations, where the healthcare services are usually limited in resources and screening and specialist services are not easily available. In clinical terms, CKD is classified into five stages depending on the severity of the disease, where stages 1–2 represent the early stage of the disease, stages 3–4 reflect moderate–severe impairment, and stage 5 is kidney failure [10]. The diagnosis and staging are based on the laboratory analysis of urinary abnormalities (excretion of proteins) and blood-based biochemical indicators of kidney activity, allowing classification of minimal kidney damage and severe renal failure [10]. Despite these well-developed clinical tools, practical risk stratification can be difficult to achieve due to the lack of reduction in the entire demographic–biochemical–clinical space that can affect the presence and progression of disease and due to the lack of important measures (such as proteinuria) or their inadequate reporting in clinical practice [11].

Artificial intelligence (AI) has become a revolutionary technology that has widespread use in a wide range of fields, such as medicine, environmental science, or transportation [12,13,14,15]. In the field of biomedicine, AI and machine learning (ML) find more applications in personalized diagnosis, risk prediction, and disease management since models can learn more complex patterns based on clinical and laboratory data [16,17,18,19]. Medical prediction is one of the tasks that machine learning approaches are most appropriate for since they can combine several variables at the same time, model nonlinear associations, and generate personalized probability instead of discrete judgment [16]. ML can be used to increase the detection performance in the context of CKD where diagnostic and prognostic decision-making is significantly dependent on biochemical and hematological markers, which can be supplemented by multi-biomarker signatures that cannot be readily identified using univariate analysis and simple rule-based reasoning [16,17]. This leads to the increased exploration of ML-based decision-support systems to aid clinicians in detecting CKD earlier and more reliably [20,21,22], particularly when clinical biomarkers gathered routinely are used.

Many ML models, despite their predictive potential, are incomprehensible, and can be viewed as black boxes, making it more challenging to promote clinical trust, acceptance, and usefulness as a model applied within the laboratory in making specific decisions [23,24,25]. Interpretability is also necessary, not only to justify individual predictions but also to ensure that model behavior is relevant to clinically meaningful biomarkers (e.g., the relationship between serum creatinine and estimated GFR and CKD status. Explainable artificial intelligence (XAI) is a leading-edge methodological framework that overcomes the weaknesses of the black-box type of predictive models by making a model output transparent and understandable. XAI can also help clinicians to critically assess the plausibility of predictions, understand the biomarkers that drive model results, and understand more intimately how predictive conclusions are made by discovering human-understandable insights into the underlying decision-making process. Such interpretability fosters trust in clinical practice, targeted and informed decision-making, and the responsible implementation of artificial intelligence in medical practice [23,24]. Shapley additive explanations (SHAP) and local interpretable model-agnostic explanations (LIME) are among the most popular XAI methods [26] that offer similar consistency in their global and local attributes based on cooperative game theory and provide instance-level explanations, respectively, making them model-agnostic and easy to interpret [23,27]. Such approaches can enhance the clinical believability of ML systems by connecting predictions to patterns of familiar biomarkers and substantiate explanation-oriented validation in clinical practices.

Guided by these requirements, the present study proposes and evaluates a falsifiable machine learning system for binary CKD prediction using clinical biomarker data, with performance assessed across two independent public datasets. The framework evaluates a large variety of ML classifiers on a standardized protocol, such as Logistic Regression (LR), Naive Bayes (NB), Decision Tree (DT), k-Nearest Neighbors (KNN), Support Vector Machine (SVM), Multi-Layer Perceptron (MLP), AdaBoost, Gradient Boosting, XGBoost, and LightGBM. We use stratified 10-fold cross-validation using out-of-fold (OOF) predictions to rigorously and clinically meaningfully assess our models to provide an accurate estimation of performance and be able to compare the performance of our models effectively. We further identify an operating threshold based on Youden’s J statistic that offers a clinically interpretable sensitivity and specificity balance and reports threshold-dependent statistics like recall (sensitivity), specificity, accuracy, F1-score, and Matthews correlation coefficient (MCC). Lastly, we combine SHAP with LIME to provide a system of complementary explainability: SHAP emphasizes the importance and directionality of biomarkers on the cohort wide, whereas LIME explains cases in individual sub-plots. Notably, considering the workflow on two datasets allows us to look at the performance strength and consistency of patterns of explanation under different feature sets and cohort features.

Unlike other studies that primarily focus on maximizing predictive performance, the present work emphasizes a clinically deployable framework that combines rigorous validation, threshold-aware decision-making, cross-dataset evaluation, and multi-level explainability to improve the transparency and reliability of CKD prediction.

The present work provides the following key findings:

1. End-to-end explainable framework, clinically oriented to predict binary CKD with routinely measured clinical biomarkers, which is intended to facilitate end-to-end screening and decision-support deployment as opposed to accuracy-only evaluation.
2. Intensive comparative evaluation of ten ML classifiers (LR, NB, DT, KNN, SVM, MLP, AdaBoost, Gradient Boosting, XGBoost, and LightGBM) through an identical preprocessing and evaluation procedure to achieve reproducibility and fair comparison.
3. Stratified 10-fold cross-validation that uses out-of-fold predictions and explicitly identifies clinically meaningful operating thresholds using the Youden J statistic, allowing the reporting of sensitivity specificity tradeoffs, Matthews correlation coefficient (MCC), and probability calibration (Brier score) instead of simply using arbitrary default cutoffs.
4. Dual-level explainability integration: Our second approach to explainability is dual-level explainability integration, combining SHAP and LIME to give not only global biomarker-level explainability but also explanatory narratives of individual patients, which make it easier to achieve transparency, auditability and clinical plausibility of model predictions.
5. The framework was tested on two samples of heterogeneous public CKD data, showing robust predictive accuracy and consistent patterns of explanation, which enhances generalizability and translational reliability.

The remainder of this paper is structured in the following way: Section 2 will conduct a literature review of related work on predicting CKD with the use of ML and XAI. Section 3 shows the methodology and talks about the suggested CKD framework. The experimental setting is described in Section 4. Section 5 describes the results and clinical implications of the same. Lastly, Section 6 is the conclusion and the future research directions.

## 2. Related Work

Over the past several years, there has been a rapid growth in the application of machine learning (ML) techniques for predicting chronic kidney disease (CKD) using routinely collected clinical and laboratory biomarkers. In comparison to traditional rule-based assessment, the ML models can leverage multivariate and nonlinear relationships between biochemical, hematological, and demographic variables to enhance early detection and risk stratification. Nevertheless, despite the promising predictive performance reported across diverse datasets and machine learning paradigms, two persistent limitations continue to hinder real-world clinical translation. First, the degree of generalization will be unclear when models are tested on only one dataset or in different validation conditions that can overstate reported performance and make it difficult to compare across studies. Second, most successful methods are based on complex ensemble models that are not interpretable enough to foster clinical trust and complicate the verification of whether the predictions are compatible with the existing kidney-function biomarkers and clinical reasoning. Due to this, explainable AI (XAI) is becoming an increasingly popular topic aimed at underpinning transparent decision-making, biomarker-level insight, and clinically plausible model behavior. Several methods to predict CKD have been investigated such as traditional classifiers, neural network classifiers and ensemble learning models. However, direct comparisons between studies are not always easy, due to variability in datasets, data preprocessing methods and validation procedures and metrics. Hence, the present study compares several representative algorithms in a common framework to offer a consistent and reproducible comparison. Recent AI literature in healthcare has highlighted the importance of not just predictive accuracy, but also the need for models to be transparent, robust, reproducible, and explainable in a way that is meaningful for clinicians in the context of clinical adoption [28,29,30]. While there are potential opportunities for XAI techniques like SHAP and LIME to contribute to interpretability and clinician trust, there are significant challenges to consider, such as stability, faithfulness, bias, and validation in real-world applications. These are especially pertinent to the prediction of CKD, wherein the output of a model needs to be correlated with clinically relevant markers and tested under strict validation conditions. Thus, XAI has emerged as a crucial line of work for the support of transparent decision-making, clinically plausible model behavior, and biomarker-level insight.

This section critically reviews the current literature on CKD prediction and explainable machine learning frameworks. Particular attention is given to dataset composition, model development strategies, validation protocols, and predictive performance, as well as to calibration, clinically meaningful operating points, and model interpretability. The reviewed studies are systematically compared to identify methodological strengths, weaknesses, and reporting inconsistencies. The synthesis of these findings enables the identification of unresolved challenges and research gaps, thereby providing the rationale for the proposed explainable CKD prediction framework.

A study by David et al., 2022 [31], compared multiple machine-learning classifiers for predicting diabetic kidney disease (DKD) using a cohort dataset of 410 instances and 18 attributes, implemented in WEKA. The authors evaluated nine classifiers (including IBK/KNN, Random Tree, Random Forest, J48, Naïve Bayes, MLP, AdaBoostM1, Hoeffding Tree, and REP Tree) and used Partition Membership Filter for preprocessing, followed by 10-fold cross-validation to assess execution time and classification performance (accuracy, kappa, MAE, RMSE, and confusion-matrix outcomes). They reported that IBK and Random Tree achieved the best overall performance with 93.6585% accuracy, kappa = 0.8731, and the lowest RMSE ≈ 0.2496, with 15 false positives and 11 false negatives in the confusion matrix. The study’s main limitation is that it focuses on a single DKD dataset with primarily accuracy/error-based reporting and no explainability (e.g., SHAP/LIME) or external validation, lowering clinical interpretability and generalization beyond the studied cohort.

An alternative study by Reddy et al. 2024 [9] suggested an explainable ML framework to forecast CKD progression (kidney failure) based on pathology data gathered periodically to reach reliably prognostic performance even when using comparatively small datasets. The analysis imitated clinically significant longitudinal characteristics age, gender, most recent eGFR, mean eGFR and eGFR slope over time and trained interpretable tree-based models (Decision Tree and Random Forest). To overcome the issue of class imbalance in the Australian cohort, the study utilized centroid cluster-based under sampling, in which K-means clustering was employed to generate representative centroids of the majority class, thereby improving class balance while preserving the underlying data structure. Discrimination was strong with internal validation of an Australian tertiary cohort (ROC-AUC = 0.94 (DT) and 0.98 (RF)) and external validation of a Japanese CKD registry (ROC-AUC = 0.88 (DT) and 0.93 (RF)) and, to increase the ability to generalize results to other populations, the authors used transfer learning with fine-tuning on 15% of the Japanese dataset. Lastly, the framework focused on clinical transparency using SHAP and LIME (along with counterfactual analysis) to describe predictions at both global and patient-specific levels to facilitate actionable interpretation of risk drivers (i.e., recent eGFR behavior and decline rate).

Arif et al., 2024 [32], suggested an explainable ML model to predict binary CKD by integrating a multi-layer perceptron (MLP) model and LiME to improve interpretability and clinical transparency. The UCI CKD dataset (400 samples, 24 predictor variables with some missing values) was used by the authors, and KNN imputation of numerical variables, mode imputation of categorical variables, and minmax scaling were used with SelectKBest (mutual information) to select the 12 most significant features. The suggested MLP was tested on 75/25 train–test split and compared to a range of classical models namely Ridge Classifier, SGD Classifier, Bernoulli Naïve Bayes, Logistic Regression, Gaussian Naive Bayes, Random Forest and Decision Tree. The results of performance were very high: the proposed model has 100% accuracy/precision/recall/F1, whereas baseline accuracies were 93% (Bernoulli NB) and 99% (SGD), with others at 95% (Ridge), (e.g., Logistic Regression 98%). Lastly, the research demonstrated instance-level LIME explanations to demonstrate the use of individual biomarkers to differentiate CKD and normal predictions to enhance the transparency of the diagnostic results. The use of one public dataset and a single hold-out split (instead of repeated CV/OOF evaluation) is a major weakness and may be inflating. Performance estimates make generalization and explanation stability less definite.

The paper by S. K. Ghosh and A. H. Khandoker, 2024 [33], suggests an ML-based model to predict CKD by categorizing patients into two groups of severity (stages 1 to 2 vs. 3 to 5), and the authors compared different baseline classifiers, such as Logistic Regression (LR), Random Forest (RF), Decision Tree (DT), and Naive Bayes (NB) to XGBoost, which worked the most successfully (accuracy 93.29%, sensitivity 91%). The authors also carried out hyperparameter search among models and fixed chosen configurations to be trained. To cope with the concerns related to black boxes they incorporated explainability frameworks based on SHAP and LIME, which not only offer global insights (feature importance and SHAP summary behavior) but also offer local, patient-specific insight. Explanations: The analysis of the explanation indicated that creatinine, HbA1C and age were the most significant factors in the XGBoost predictions, which may be a sign of the clinical significance of a small biomarker-driven model to identify CKD in time.

One such deep learning model is the study by G. Hegde, P. D. Shenoy, and A. Canchi, [34] which explicitly integrates feature selection and explainability in a hybrid approach termed MHMXAI. The authors believe that most feature selection models are optimized without interpretability and that XAI methods like SHAP and LIME are explanatory but not meant to search for optimal subsets. Thus, MHMXAI is proposed to fulfill both purposes by seeding metaheuristic and hybrid metaheuristic search (ESA, GSR, and TEO) with SHAP and LIME importance scoring to prioritize features and drop approximately half of the least relevant variables for downstream modeling. The framework consists of three stages–data preprocessing (eGFR calculation, CKD staging, and data enhancement), feature selection, and model development—and was evaluated on a hospital cohort from Bangalore Hospital, India. For prediction, the authors benchmarked eight DL models involving RNN, LSTM, BiLSTM, DNN, CNN, GRU, and BiGRU across various FS configurations and observed that CNN persistently works better, at an accuracy of about 98–99.5 percent, and the CNN trained on MHMXAI identified features was the strongest solution found by Friedman post hoc testing and Nemenyi post hoc testing. The authors themselves also acknowledge several limitations that affect generalizability, including the predominant use of blood and urine characteristics without major comorbidities, single-site data, limited EHR feasibility, and data quality issues. Despite these limitations, they propose MHMXAI as a scalable and clinically interpretable approach for CKD staging models.

H. Iftikhar, A. F. Hashem, M. Qureshi, and P. C. Rodrigues [35] developed a study that examined the detection of early-stage CKD using a clinically annotated binary dataset (CKD vs. non-CKD), sampled at the Burner Medical Complex (BMC) and tested individual and ensemble machine learning methods to support clinical decision-making. They compared a broad range of classical ML models covered, Logistic Regression, LDA, QDA, Ridge Classifier, Naive Bayes, KNN, Decision Tree, Random Forest and SVM, and even discussed strategies of ensemble (voting and stacking) with a well-organized preprocessing workflow and feature selection to minimize noise and enhance clinical usability. Performance was measured with the help of standard classification measurements (accuracy, precision, recall, F1-score, and ROC-AUC), and robustness and generalization were checked with the help of a 5-fold and 10-fold stratified cross-validation. The findings indicated that there were always high levels of discrimination towards CKD screening and that the Random Forest model generated the best results in resampling schemes (reported accuracies ≈ 91.58% (5-fold) and 90.53% (10-fold)) and the ensembles also generated competitive results and offered greater stability, which supports the argument that ensemble learning could be more robust in providing decision support in a real-world environment where variability occurs. The current paper also highlighted practical clinical applicability in the context of low-resource and rural telemedicine by making use of a limited set of routinely available variables, and it pointed to the importance of interpretability and feature relevance such as a feature selection phase of transforming ML outputs into practicable results. The authors, however, recognized several limitations to clinical generalizability: the study is cross-sectional, includes one center, and does not include sufficiently important sociodemographic variables, which should be overcome in subsequent studies with larger, multi-center, longitudinal cohorts and more meaningful clinical and lifestyle, and genetic factors to enhance external validity and real-world implementation.

The study by Kim and Woo [36] developed a deep learning-based mortality prediction model for CKD patients using a large national administrative dataset from the Korea National Hospital Discharge In-depth Injury Survey (KNHDIS), focusing deliberately on general patient and healthcare utilization characteristics rather than laboratory biomarkers to improve practicality in primary and secondary care settings. The cohort included 12,680 CKD patients (ICD-10 N18.X) from 2016 to 2021, split temporally into training and validation (2016–2020; *n* = 10,498) and an independent test set (2021; *n* = 2182) to simulate forward-looking deployment. The workflow applied LASSO regression for feature selection compared with logistic regression, identifying eight key predictors such as length of stay (LOS), emergency admission, age, Charlson Comorbidity Index (CCI), region, hospital bed-size category, and insurance, then trained a multi-layer neural network with two hidden layers, dropout of 50%, ReLU, and Adam and benchmarked it against Random Forest and XGBoost. The final model achieved AUC = 0.83 with accuracy ≈ 96.83%, and on the withheld 2021 test set it reported accuracy = 96.84% (loss = 0.1207), outperforming the compared ML baselines in AUC (RF 0.79; XGBoost 0.71). A key limitation for translation to CKD diagnosis and biomarker-driven prediction is that the task targets mortality prognosis rather than CKD detection, uses general characteristics instead of laboratory biomarkers, and does not apply SHAP or LIME. The authors explicitly note the need for external validation and interpretability tools in future work.

The study by J. Huang, L. Li, M. Hou, and J. Chen [37] proposed an integrated clinical decision framework that jointly optimizes model performance and interpretability for CKD risk stratification by combining Optuna (Tree-structured Parzen Estimator, TPE) hyperparameter optimization with SHAP explanations. The authors emphasize that prior CKD studies often treat tuning and interpretability as separate steps; in contrast, their pipeline unifies data-quality handling (e.g., KNN-based imputation), bias-aware evaluation via macro-averaged metrics to reduce majority class favoritism, and post hoc explainability to support clinical plausibility. Empirically, the Optuna-optimized XGBoost achieved 92.4% accuracy, 91.9% F1, and 97.7% ROC-AUC, significantly outperforming a large set of baselines while reducing optimization time versus grid search—a key advantage for resource-constrained settings. SHAP analysis identified CKD stage, albumin–creatinine ratio, and eGFR as the most influential predictors, aligning with clinical guidelines and supporting interpretability. The study acknowledges limitations (single-region, cross-sectional data) and recommends multi-center, longitudinal validation as future work.

A summary of prior CKD-related machine learning studies and comparison with the proposed framework, highlighting task definition, data source, validation rigor, explainability, and whether a clinically oriented operating threshold is explicitly reported is given in Table 1.

Previous works have demonstrated ML’s ability to predict CKD, yet several limitations hinder clinical application of such algorithms in clinical practice. First, most studies take a single data source, which limits the evaluation of the robustness and generalizability of the results reported. Second, the validation protocols used in different studies are also quite diverse, from single train–test splits to using other cross-validation protocols, making it difficult to evaluate the different studies fairly and potentially giving an optimistic estimate of the performance. Third, numerous studies focus on discrimination metrics (e.g., accuracy, ROC-AUC) and pay little attention to the selection of operating points, particularly the crucial balance between sensitivity and specificity, which is essential in screening applications. Finally, while explainable AI methods are gaining traction, only a handful of studies offer both a rigorous evaluation of the model along with both global and local interpretability.

To overcome these limitations, the current study implements a clinically oriented and explainable machine learning model for CKD prediction. The proposed approach differs from previous studies by systematically testing 10 machine learning models in two independent datasets for the task of CKD, with a consistent preprocessing and validation pipeline. Stratified 10-fold cross-validation and out-of-fold prediction are used for performance assessment, and Youden’s J statistic is used to determine clinically relevant decision thresholds. Moreover, SHAP is designed to be integrated with LIME to give population-level explanation of biomarkers and patient-specific explanation. All of these methodological elements provide a more transparent, reproducible, and clinically interpretable approach to CKD prediction than is often reported in the literature.

## 3. Materials and Methods

**Figure 1:**
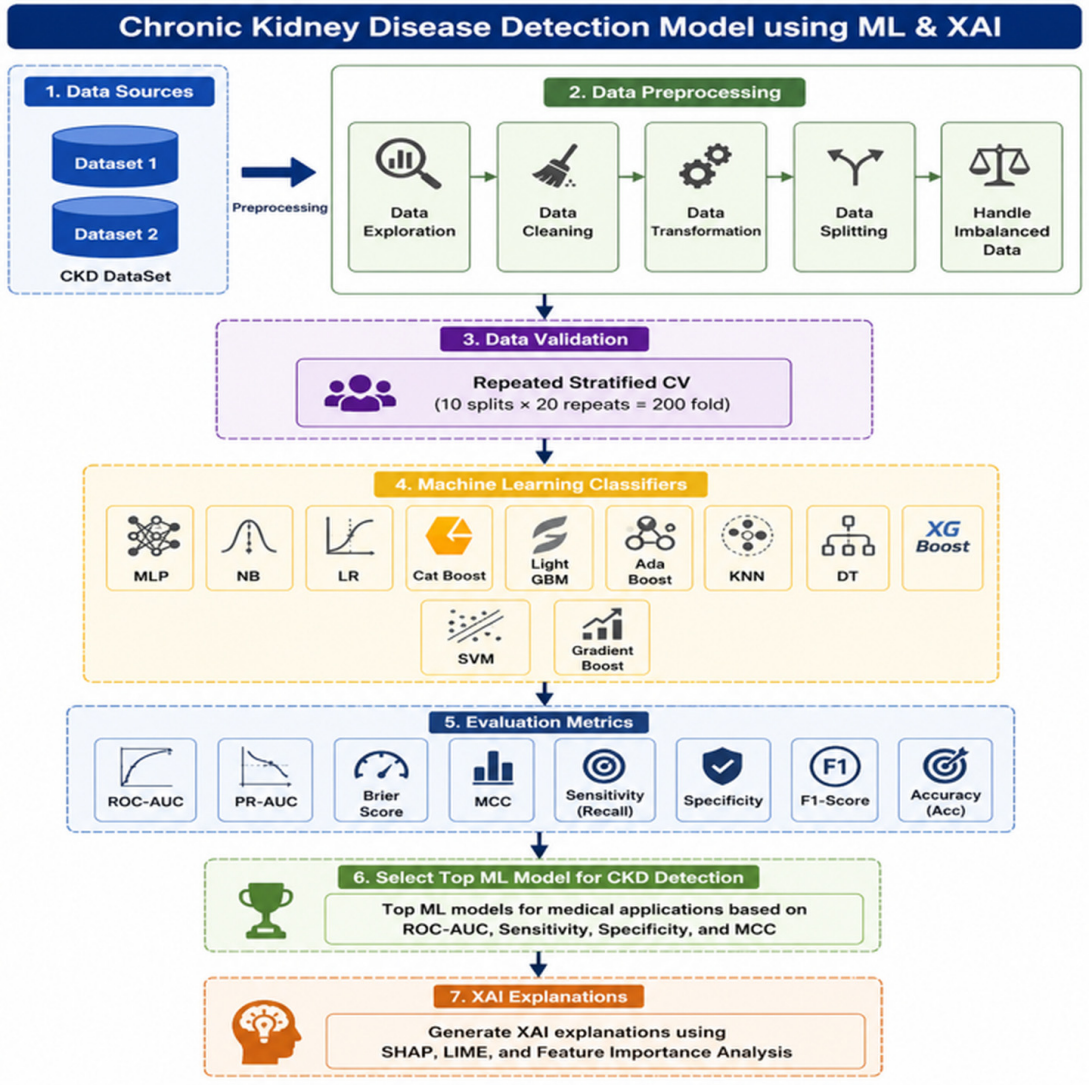
Flowchart of the proposed framework.

In this section, the methodological pipeline that was employed in developing and assessing the proposed explainable machine learning framework to predict binary CKD based on clinical biomarkers is described. Initially, this section introduces the two publicly available datasets used for CKD prediction. The subsequent subsection outlines the preprocessing pipeline applied to enhance data integrity, handle missing values, standardize variables, and maintain consistency across models. The assessed machine learning classifiers and experimental setup are then described in detail. Model performance is evaluated using stratified 10-fold cross-validation, while out-of-fold (OOF) predictions are generated to provide robust and unbiased estimates of predictive performance. Furthermore, Youden’s J statistic is employed for threshold optimization, allowing the identification and reporting of clinically relevant operating points and their variability across validation folds. Finally, the explainability component of the framework is presented, whereby SHAP and LIME are used to provide complementary interpretations of model predictions at both the global and patient-specific levels. While SHAP identifies the overall importance of clinical biomarkers across the dataset, LIME explains the factors influencing individual predictions. Collectively, these components form a unified workflow that combines robust predictive modeling with interpretable, biomarker-driven insights, thereby enhancing clinical relevance and supporting informed decision-making. Figure 1 demonstrates a general flowchart of framework of the proposed methodology.

### 3.1. Datasets

In this study, two publicly available CKD-related datasets were utilized to evaluate the proposed framework and to examine its robustness across different feature sets and cohort characteristics.

#### 3.1.1. Dataset 1

The first dataset used in this research was obtained from the UCI Machine Learning Repository [38]. It contains 400 instances described by 24 features and one class (demographic, clinical, and laboratory attributes). Each sample includes 24 features variables, 11 numerical and 13 nominal and categorical, and a binary target label indicating CKD or non-CKD. Before any balancing procedures, the dataset comprised 400 samples with 24 features, and the class distribution was 250 (class = CKD) and 150 (class = non-CKD). Table 2 shows the dataset list of features.

#### 3.1.2. Dataset 2

The second dataset was obtained from a publicly available Kaggle source [39] and contains comprehensive health records for 1659 patients related to chronic kidney disease (CKD). It includes a broad set of attributes covering demographics, lifestyle factors, medical history, clinical measurements, medication usage, symptoms, quality-of-life indicators, environmental exposures, and health behaviors, with each record uniquely indexed by a Patient ID (and a confidential field indicating the responsible doctor). Before any balancing procedures, the dataset comprised 1659 samples with 51 predictors, and the class distribution was 1524 (class = 1) and 135 (class = 0). Table 3 shows the dataset list of features.

### 3.2. Dataset Preprocessing

To have resilient and repeatable experimentation, we applied a formal preprocessing pipeline comprising two specialized modules, medical data cleaner and medical preprocessor, which are used to perform fold-safe transformations and fold-safe artifact processing, respectively, to avoid data leakage cross-validation. Before the models’ training, both datasets were checked for missing values, inconsistent encodings, and data quality problems. For Dataset 1, the missing values were mostly replaced with the placeholder “?” which was then transformed to the standard missing value (NaN). Overall, there were 1012 missing values in Dataset 1, which is about 10.12% of all cells of the dataset. The highest missingness rates were observed in red blood cells (rbc: 38.00%), red blood cell count (rbcc: 32.75%), white blood cell count (wbcc: 26.50%), potassium (pot: 22.00%), sodium (sod: 21.75%), packed cell volume (pcv: 17.75%), pus cell (pc: 16.25%), hemoglobin (hemo: 13.00%), sugar (su: 12.25%), specific gravity (sg: 11.75%), albumin (al: 11.50%), and blood glucose random (bgr: 11.00%). Blood urea, serum creatinine, blood pressure, age and some binary clinical parameters had lower missingness. The second dataset (Dataset 2) did not have any missing data but was run through the same quality-control pipeline to ensure uniformity of the datasets.

Information leakage was addressed by using a loop for CV, within which the missing value imputation and feature transformations were performed. Numerical variables were imputed using KNN imputation, and categorical variables were imputed using the most frequent category in the training data for each training fold. After imputation, numerical variables were scaled according to the standard scaling method while categorical variables were encoded with the ordinal encoding method and unknown category handling. The learned parameters for imputation, scaling and encoding were then applied to the validation fold. This way, no information was used in the validation data during preprocessing, which gave reliable out-of-fold predictions and more trustworthy model evaluation. The pipeline had five consecutive steps such as exploration data analysis, cleaning, transformation, stratified splitting, and mitigation of class imbalance.

#### 3.2.1. Data Exploration

Prior to model training, exploratory checks were performed to characterize data quality and identify potential issues affecting downstream learning. Feature-level missingness was quantified for each variable j by computing the missing count *mj* and missing rate *rj* as in Equation (1):

(1) $$m_{j}=\sum_{i=1}^{N}I(x_{ij}=NaN),r_{j}=\frac{m_{j}}{N},$$

where *N* is the number of samples and $I\left(\cdot \right)$ is the indicator function. The ranked missingness summary and visualization were saved for reporting.

In addition, numeric features were screened for extreme values using z-scores as in Equation (2):

(2) $$z_{ij}=\frac{x_{ij}-\mu_{j}}{\sigma_{j}},$$

and potential outliers were flagged when $|z_{ij}|\;>\;\tau$ (default τ=3); these outlier statistics were recorded for transparency rather than being forcibly removed in the provided implementation.

#### 3.2.2. Data Cleaning

Cleaning operations were applied to standardize labels, handle missing value tokens, and harmonize categorical entries. The binary target label was normalized and mapped into {0,1} using a deterministic mapping f(⋅), as in Equation (3):

(3) $$y_{i}=f(y_{i}^{\mathrm{raw}})=\left\{\begin{array}{cc} 1, & y_{i}^{\mathrm{raw}}\in [\mathrm{ckd},\mathrm{yes}]\\ 0, & y_{i}^{\mathrm{raw}}\in [\mathrm{notckd},\mathrm{no}] \end{array}\right.$$

and the pipeline verifies that exactly two unique classes exist. Common missing-value placeholders (e.g., “?”, “NA”, “N/A”, “null”, empty strings) were replaced by NaN using Equation (4).

(4) $$x_{ij}\leftarrow \left\{\begin{array}{cc} NaN, & x_{ij}\in \Omega \\ x_{ij}, & \mathrm{otherwise} \end{array}\right.$$

where Ω denotes the set of missing tokens.

Finally, feature names were normalized as a consistent scheme to ensure stable references across the pipeline.

#### 3.2.3. Data Transformation

Transformations were performed in a type of aware manner after automatically identifying numeric and categorical feature subsets as in Equation (5):

(5) $$N=\left\{j:\mathrm{feature}\;j\;\mathrm{is}\;\mathrm{numeric}\right\},C=[1,\ldots ,p]\setminus N$$

Low-cardinality features (≤10 unique values) were optionally coerced to be numeric when feasible. Within each training fold, numeric variables were imputed (KNN-based in the preprocessor) and categorical variables were imputed using the most frequent category. A standard KNN-imputation formulation can be expressed as in Equation (6):

(6) $${\hat{x}}_{ij}=\frac{\sum_{n\in N_{K}(i)}w_{in}\;x_{nj}}{\sum_{n\in N_{K}(i)}w_{in}},w_{in}=\frac{1}{d(x_{i},x_{n})+\epsilon },$$

where $N_{K}(i)$ is the set of KNNs and $d(x_{i},x_{n})$ is a distance function. Numeric features were standardized using training-fold statistics as in Equation (7):

(7) $$x_{ij}^{\left(\mathrm{scaled}\right)}=\frac{x_{ij}-\mu_{j,\mathrm{tr}}}{\sigma_{j,\mathrm{tr}}},$$

and categorical features were encoded using an ordinal encoding map $\psi_{j}\left(\cdot \right)$ with unknown categories mapped to a reserved code during validation as in Equation (8):

(8) $$x_{ij}^{\left(\mathrm{enc}\right)}=\psi_{j}(x_{ij}),x_{ij}\notin V_{j}\Rightarrow x_{ij}^{\left(\mathrm{enc}\right)}=-1.$$

#### 3.2.4. Data Splitting

Model evaluation followed a stratified cross-validation design to preserve the original class proportions in each fold. Specifically, the dataset *D* was partitioned into *K = 10* stratified folds $\{D^{\left(t\right)}{\}}_{t=1}^{K}$ such that in Equation (9):

(9) $$\frac{|[i\in D^{\left(t\right)}:y_{i}=1]|}{|D^{\left(t\right)}|}\approx \frac{|[i\in D:y_{i}=1]|}{|D|},\forall t.$$

Critically, preprocessing was performed in a leakage-free manner by fitting the preprocessor only on the training subset of each fold and then transforming both training and validation partitions using the same learned parameters as in Equation (10):

(10) $$\theta^{\left(t\right)}=Fit\left(X_{\mathrm{tr}}^{\left(t\right)}\right),X_{\mathrm{tr}}^{{\left(t\right)}^{\prime }}=Transform\left(X_{\mathrm{tr}}^{\left(t\right)};\theta^{\left(t\right)}\right),X_{\mathrm{va}}^{{\left(t\right)}^{\prime }}=Transform\left(X_{\mathrm{va}}^{\left(t\right)};\theta^{\left(t\right)}\right).$$

#### 3.2.5. Handle Imbalanced Data

To mitigate class imbalance, the pipeline optionally applies SMOTE-based oversampling. Because SMOTE requires fully numeric and non-missing inputs, the code first performs a temporary NaN-aware filling step: numeric features are filled by the median and categorical features by the mode (with safe fallbacks), then categorical values are label-encoded to integers prior to resampling. SMOTE generates synthetic minority samples by interpolating between a minority sample x and one of its k-nearest minority neighbors $x_{\mathrm{nn}}$ as in Equation (11)

(11) $$x_{\mathrm{new}}=x+\lambda (x_{\mathrm{nn}}-x),\lambda ∼U(0,1),$$

with *k* adaptively adjusted based on the minority class size; if SMOTE fails, the pipeline falls back to random oversampling. After resampling, categorical features are inversely transformed back to their original labels when possible and data types are restored.

### 3.3. Machine Learning Models

To fairly and robustly assess the ability of the ML classifiers to detect CKD, a diverse set of classifiers were chosen across learning paradigms that are often applied in structured clinical prediction problems [40]. Logistic Regression and Naïve Bayes were chosen as simple, interpretable statistical baseline classifiers and Decision Tree, KNN, and SVM as tree-based [41], distance-based, and kernel-based classifiers. The incorporation of MLP was used as a measure to evaluate the potential of neural modeling to capture nonlinear interactions between the various biomarkers of CKD. Furthermore, ensemble boosting models (AdaBoost, Gradient Boosting, XGBoost, and LightGBM) were considered [42], as they have been found to be very suitable for tabular biomedical data and can model complex relationships among demographic, clinical, and laboratory variables effectively. These algorithms are compared within the same preprocessing, validation, threshold-selection and explainability framework, allowing the study to avoid any dependence on a certain model architecture and fair assessment of the predictive performance, stability and clinical interpretability. The goal was not to present a new model architecture but to determine the “best” one that is reliable and explainable in a clinically oriented evaluation setting in the context of binary prediction of CKD.

### 3.4. Selection of the Top ML Model for CKD Detection and XAI Explanations

After benchmarking the ten candidate classifiers, the top model was selected based on a clinically oriented, cross-validated out-of-fold (OOF) evaluation rather than a single split, to ensure an unbiased estimate of real-world performance. Let ${\hat{p}}_{i}$ denote the OOF-predicted probability of CKD for sample *i*. Using these OOF probabilities, an operating threshold $\tau^{*}$ was determined by maximizing Youden’s J statistics, which provides a balanced tradeoff between sensitivity and specificity as in Equation (12):

(12) $$J\left(\tau \right)=Sensitivity\left(\tau \right)+Specificity\left(\tau \right)-1,\tau^{*}=arg\underset{\tau \in \left[0,1\right]}{max}J\left(\tau \right)$$

Sensitivity and specificity at threshold τ are computed from the confusion matrix terms $\left(TP,TN,FP,FN\right)$ as:

$$Sensitivity\left(\tau \right)=\frac{TP\left(\tau \right)}{TP\left(\tau \right)+FN\left(\tau \right)},Specificity\left(\tau \right)=\frac{TN\left(\tau \right)}{TN\left(\tau \right)+FP\left(\tau \right)}$$

The predicted class label is then obtained as in Equation (13):

(13) $${\hat{y}}_{i}=\left\{\begin{array}{cc} 1, & {\hat{p}}_{i}\geq \tau^{*},\\ 0, & {\hat{p}}_{i}<\tau^{*}. \end{array}\right.$$

The top model was selected by prioritizing discrimination and clinical utility using OOF metrics, including ROC-AUC, PR-AUC, and Brier score, while also reporting threshold-dependent performance at $\tau^{*}$ namely accuracy, F1-score, MCC, sensitivity, and specificity. Accuracy and F1-score are defined as:

$$Accuracy=\frac{TP+TN}{TP+TN+FP+FN},F1=\frac{2TP}{2TP+FP+FN}$$

and the Matthews correlation coefficient (MCC) as in Equation (14):

(14) $$MCC=\frac{TP\cdot TN-FP\cdot FN}{\sqrt{\left(TP+FP)(TP+FN)(TN+FP)(TN+FN\right)}}$$

To assess probability calibration, the Brier score was computed from the OOF probabilities in Equation (15):

(15) $$Brier=\frac{1}{N}\sum_{i=1}^{N}{\left({\hat{p}}_{i}-y_{i}\right)}^{2}$$

Following model selection, explainability was incorporated to ensure that predictive decisions are clinically interpretable and biomarker-consistent. Two complementary XAI methods were applied: SHAP for global and local feature attribution and LIME for instance-level [43], model-agnostic explanations. For SHAP, the prediction for a sample x is decomposed into additive feature contributions in Equation (16):

(16) $$f\left(x\right)=\phi_{0}+\sum_{j=1}^{p}\phi_{j}$$

where $\phi_{0}$ is the expected model output (baseline) and $\phi_{j}$_j quantifies the marginal contribution of feature j to the prediction. For LIME, an interpretable surrogate model g(⋅) (typically sparse linear) fits locally around x by minimizing in Equation (17):

(17) $$arg\;\underset{g\in G}{min}\left(f,g,\pi_{x}\right)+\Omega \left(g\right)$$

where f is the original model, $\pi_{x}$ weights perturbed samples by proximity to x, L measures fidelity between f and g, and Ω(g) penalizes complexity to promote interpretability. Together, SHAP and LIME provide complementary evidence by identifying both consistent global biomarkers influencing CKD detection and patient-specific drivers of individual predictions, supporting clinical plausibility and trust in the selected model.

In summary, the proposed evaluation protocol is a combination of stratified 10-fold cross-validation followed by out-of-fold probability aggregation to generate objective estimations of performance and a clinically based operating point by choosing the decision threshold that maximizes Youden’s J statistic. It is a design allowing uniform reporting of both discrimination and calibration (ROC-AUC, PR-AUC, and Brier score) as well as threshold-dependent clinical measures (sensitivity, specificity, accuracy, F1-score, and MCC), and it also reports on the computational feasibility using fold-wise runtime statistics. Continuing this strict and reproducible protocol, the following section shows the experimental setting, implementation setting, configuration options, parameter options, and the experimental practical configuration, which was employed in implementing all experiments on the two datasets.

## 4. Experimental Setting

All of the experiments were conducted in Python 3.13 on a workstation with an Intel core i7 (3.2 GHz) processor, 16 GB RAM, and Windows 11 (64-bit). The Scikit-learn library was used to execute the implementations of the classical ML models, and LightGBM and XGBoost were used to execute the implementations of advanced ensemble and gradient boosting classifiers other libraries are in favor of. NumPy(2.1.3), Pandas (2.2.3), Matplotlib (3.10.0), and Seaborn (0.13.2) were used as data handling, analysis and visualization. To validate the evaluated models against one another, all ML classifiers were assessed under identical stratified 10-fold cross-validation splits and preprocessing conditions. This paired evaluation design ensured that each model was trained and tested on the same fold partitions, enabling direct comparison across algorithms. In addition to reporting discrimination and threshold-based metrics, fold-wise results were compared statistically to examine whether observed differences between models were consistent across validation folds. Multiple-comparison correction was applied when comparing several classifiers to reduce the risk of false-positive significance claims. Table 4 and Table 5 display the experimental parameter settings and hyperparameter settings of the classifiers, respectively.

Since the data used to evaluate the predictive performance of the models are of cross-sectional form, each data point was assumed to be an independent record at the level of the individual. There was no follow-up at any time point of the same patient because the dataset did not include longitudinal studies. Hence, all predictions were made from single-time point measures. Consequently, a stratified 10-fold cross-validation was carried out at the patient/sample level, and any non-predictive identifiers, when present, were excluded from the feature matrix prior to model training. Additionally, every preprocessing step was learned only on the training part of each fold, then later used on the matching validation partition. Thus, the whole point was to reduce the chance of information leakage, as much as possible. To have a fair comparison, all baseline and advanced models were evaluated under identical experimental conditions, including the same preprocessing pipeline, stratified 10-fold cross-validation partitions, random seed, imbalance-handling procedure, threshold-selection strategy, and performance metrics.

## 5. Results and Discussion

The proposed CKD detection framework was systematically tested within the preprocessing pipeline outlined and a stratified 10-fold validation scenario using a wide range of ML classifiers, namely LightGBM, XGBoost, Gradient Boosting, AdaBoost, Support Vector machine (SVM), k-Nearest Neighbors (KNN), Multi-layer perceptron (MLP), Logistic Regression (LR), Decision Tree (dt) and Naive Bayes (NB). In order to define the model generalizability in a thorough manner, two datasets have been chosen for experiment purposes. Therefore, the Results and Discussion Section will be split into two subsections, based on results obtained from different datasets. Some models’ results were near the ceiling of prediction performance; they should be interpreted according to the properties of the datasets that were evaluated. The several highly informative clinical biomarkers, such as serum creatinine, glomerular filtration rate (GFR), albuminuria and related laboratory measurements, are strongly associated with chronic kidney disease (CKD) and have a good discriminatory ability between CKD and non-CKD cases. Model assessment was performed using 10-fold cross-validation with out-of-fold (OOF) predictions to avoid overfitting within the data. Moreover, preprocessing and class-balancing techniques were performed only during the training folds, avoiding any information leakage. The results were also found to be consistent in two independent datasets with different feature compositions and cohort characteristics, indicating that the observed performance was based on the true ability to distinguish between the two biomarker types and not memorization.

This study adopted Youden’s J statistic to determine the optimal operating threshold because it provides an objective balance between sensitivity and specificity. However, threshold selection is inherently application-dependent, and alternative approaches such as fixed probability thresholds, F1-score optimization, precision–recall-based thresholding, cost-sensitive decision rules, or decision-curve analysis may be preferred in settings where minimizing false negatives or false positives carries different clinical consequences.

### 5.1. Dataset 1

**Figure 2:**
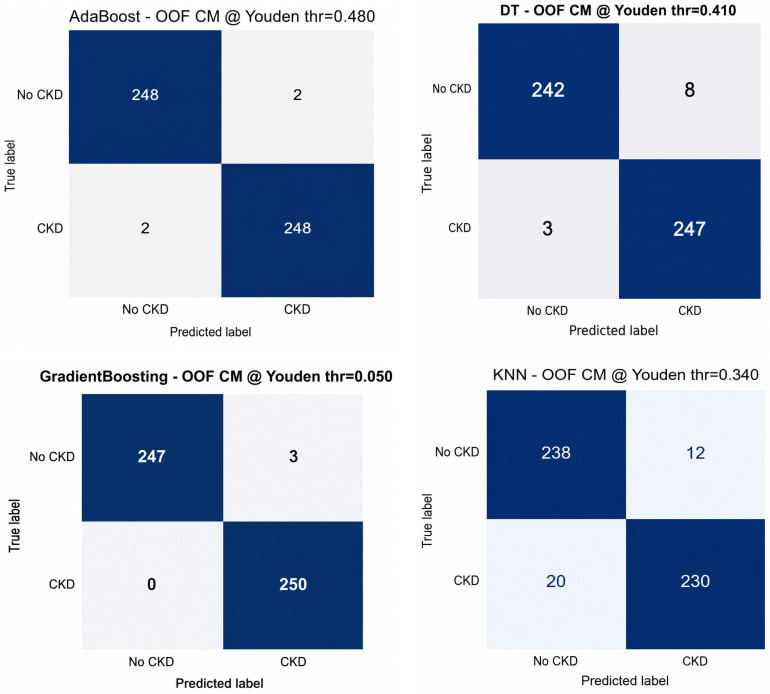

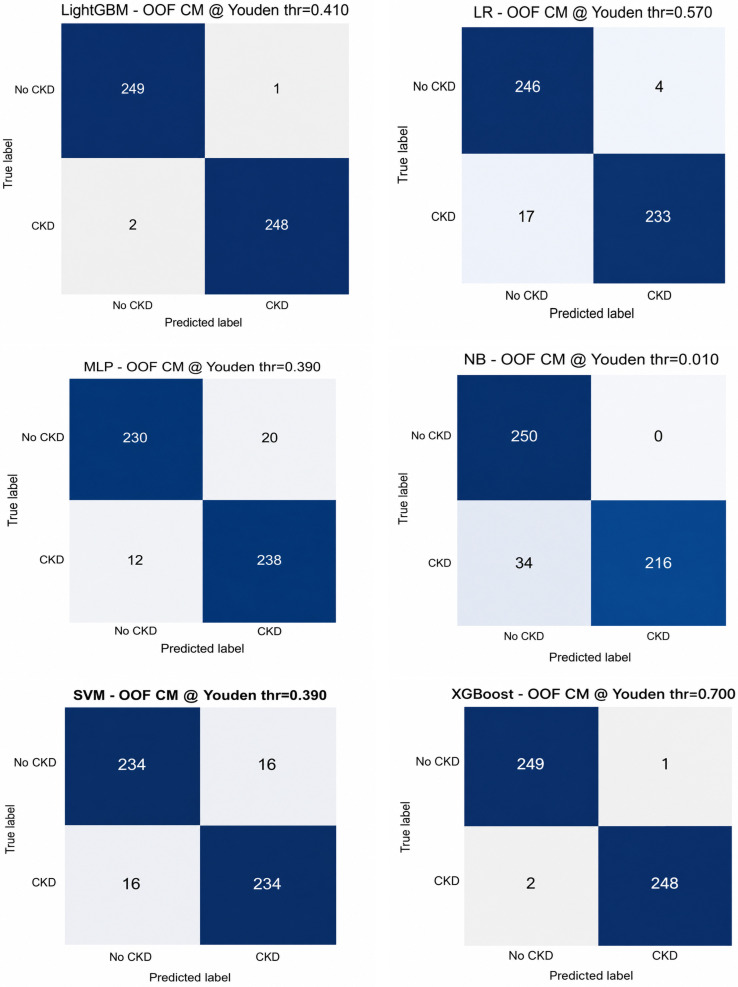
Confusion matrix of Dataset 1.

In this subsection, we report and analyze the experimental results obtained on Dataset 1 to investigate the efficacy and clinical applicability of the proposed CKD detection pipeline. Summary of the performance is done with the help of both threshold-independent discrimination and reference to the clinically oriented outcomes which are threshold-based, and as a result the best Youden threshold is considered to maintain a balance between sensitivity and specificity. Figure 2 shows a confusion matrix of each classifier at this operating point which would give a clear picture of true positives, true negatives, false positives, and false negatives in CKD and non-CKD classification. Taken together, these findings explain the relative advantages and restrictions of the considered models and assist in determining the most consistent strategy to be used in this dataset.

**Figure 3:**
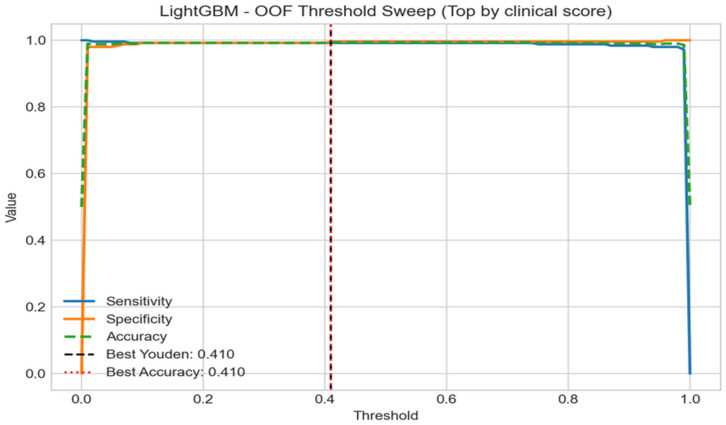
OOF threshold sweep for the top-ranked model (LightGBM).

Table 6 shows the performance comparison of each of the assessed ML models on Dataset 1 under the 10-fold CV/OOF protocol. The operating point which is clinically oriented is defined as the OOF best Youden threshold, and the results of the CV are presented in terms of the meaning with a standard deviation per fold. Lower training time is associated with the fact that the Brier score is better calibrated and yields more efficient computation. In general, the findings indicate that the gradient boosting family obtains the best results as LightGBM leads the list in combining almost-ceiling discrimination (ROC/AUC/PR-AUC) with the most balanced sensitivity and specificity tradeoff at the Youden threshold followed by XGBoost and Gradient Boosting. Even though classical baselines, including Decision Tree and Logistic Regression, are still competitive in terms of their MCC and/or sensitivity, the drop in their performance is significant in comparison to boosting methods. Incorporating these results, Figure 3 shows the OOF threshold sweep of the most ranked model (LightGBM) with sensitivity, specificity, and accuracy curves being plotted against candidate thresholds and the optimum Youden threshold being displayed to achieve a balanced tradeoff between sensitivity and specificity, as opposed to using a set default threshold (e.g., 0.50). This consideration is especially important in CKD screening, where the choice of decision threshold directly influences the false-negative to false-positive ratio at deployment. Together, Table 6 and Figure 2 establish that LightGBM achieves the best balance among screening effectiveness, error control, and strong agreement, rendering it a good candidate for the explainability analysis of Dataset 1.

**Figure 4:**
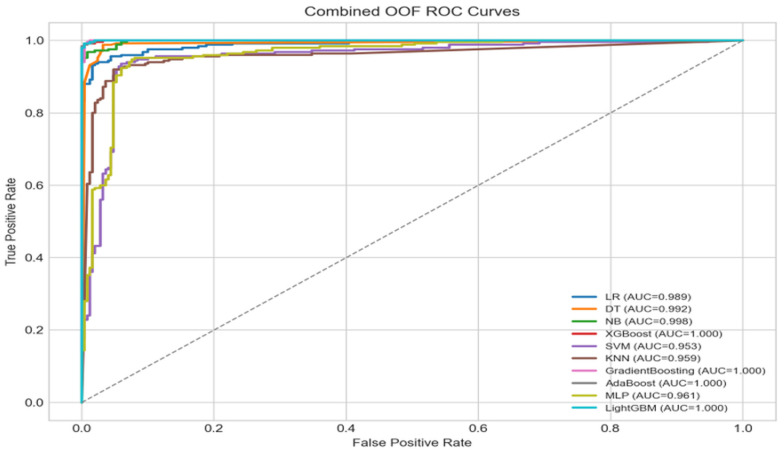
OOF ROC curves of all classifiers.

A comparison of the OOF ROC curves of all classifiers on Dataset 1 is shown in Figure 4. The ROC curve shows the tradeoff between the sensitivity (true-positive rate) and the false-positive rate over all possible thresholds. The figure shows that the gradient boosting models specifically LightGBM, XGBoost, Gradient Boosting and AdaBoost are close to perfect discrimination. clustered around the top-left corner and the highest values in the AUC, which means that separability is excellent among CKD and non-CKD samples. Conversely, the traditional classification tools like SVM, KNN, and MLP have lower AUC and a less steep increase at low false-positive rates, which indicates weaker discrimination with severe screening restrictions.

**Figure 5:**
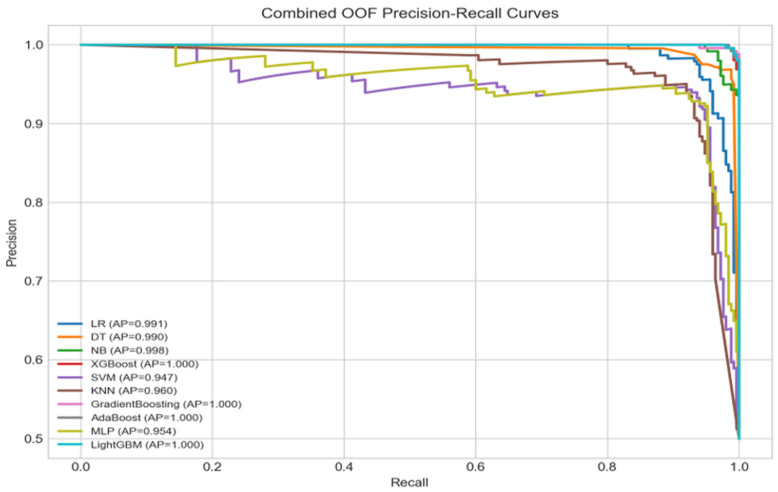
OOF precision–recall (PR) curves for all classifiers.

Figure 5 reports the combined OOF precision–recall (PR) curves, which are particularly informative in clinical screening because they emphasize performance on the positive class (CKD) by relating precision (positive predictive value) to recall (sensitivity). The PR results are consistent with the ROC analysis: the top boosting models maintain very high precision across a broad recall range, yielding the highest average precision (AP). By comparison, models such as SVM, KNN, and MLP exhibit a more noticeable precision decline as recall approaches 1.0, implying a higher false-positive burden when attempting to maximize sensitivity. Overall, Figure 4 and Figure 5 confirm that LightGBM and other boosting methods provide the most robust threshold independent discrimination for CKD detection on Dataset 1.

**Figure 6:**
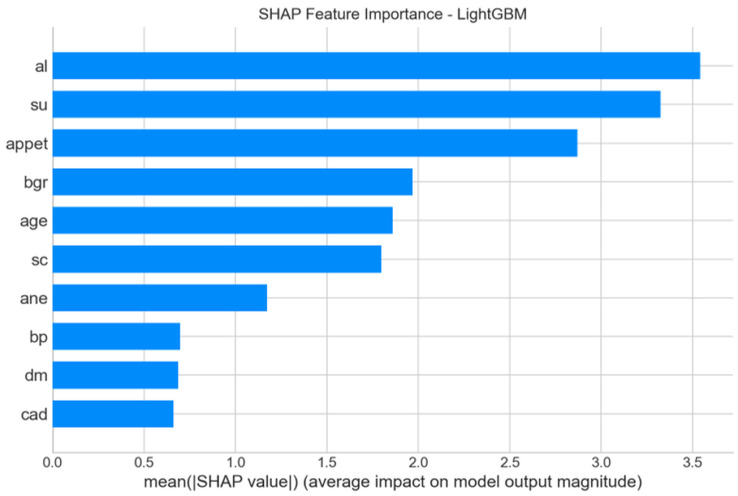
SHAP feature importance: LightGBM.

Figure 6 presents the global SHAP feature importance. As shown, LightGBM’s decision processes follow a clinically coherent pattern, primarily drawing from kidney-related and cardiovascular metabolic indicators. Among these, albumin (al) and sugar (su) emerge as the top contributors, both of which are key urine markers. Some abnormalities which are routinely checked during kidney disease screening and staging procedures are highlighted. These include serum creatinine (sc), blood glucose level (bgr), and age, which represent the overall metabolic impact on the body as well as its renal filtering capability. Significantly, the importance of variables such as appetite (appet) and anemia (ane), which commonly occur due to decreased renal functioning and critical illnesses, has been emphasized in this model along with some other risk factors including blood pressure (bp), diabetes mellitus (dm), and coronary artery disease (cad). Taken together, the relative importance of these features demonstrates that the important results obtained through this model are not mere statistical outcomes but clinically relevant.

**Figure 7:**
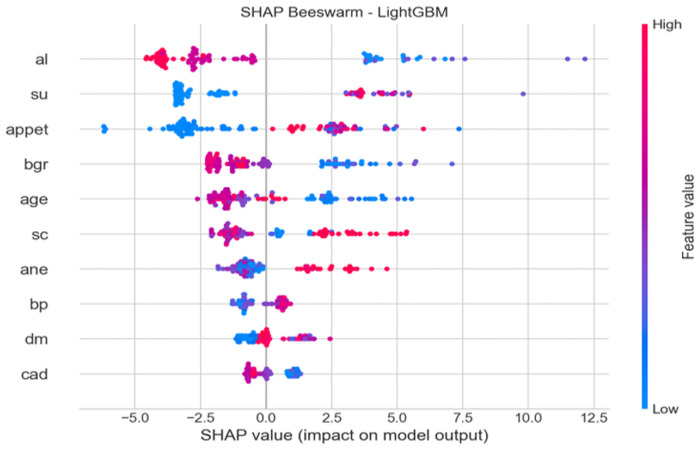
SHAP beeswarm—LightGBM.

The SHAP beeswarm plot of LightGBM is presented in Figure 7. It represents an additional example of the influence of the features’ value on prediction changes between two cohorts, i.e., low versus high biomarker value. A patient is represented by a point, and the horizontal dispersion is used to measure the extent to which that feature shifts the model output towards the CKD class (positive SHAP) versus non-CKD (negative SHAP) by the label encoding that occurs in the pipeline. One important clinical observation is that a biomarker acting in a variable manner in different patients may be the same biomarker. Whether it is due to the range of its values or relating to other variables precisely is a reality that clinicians must deal with in practice (e.g., borderline lab values of low-risk patients vs. that same value in high-risk, multimorbidity patients). Since the presence of albumin (al) and sugar (su) occurs so often, the results of the urine tests cannot be seen as mere binary triggers. They are key influencers that establish the certainty of the model. By determining the classification of chronic kidney disease, the model gets to the very core of clinical decision-making.

**Figure 8:**
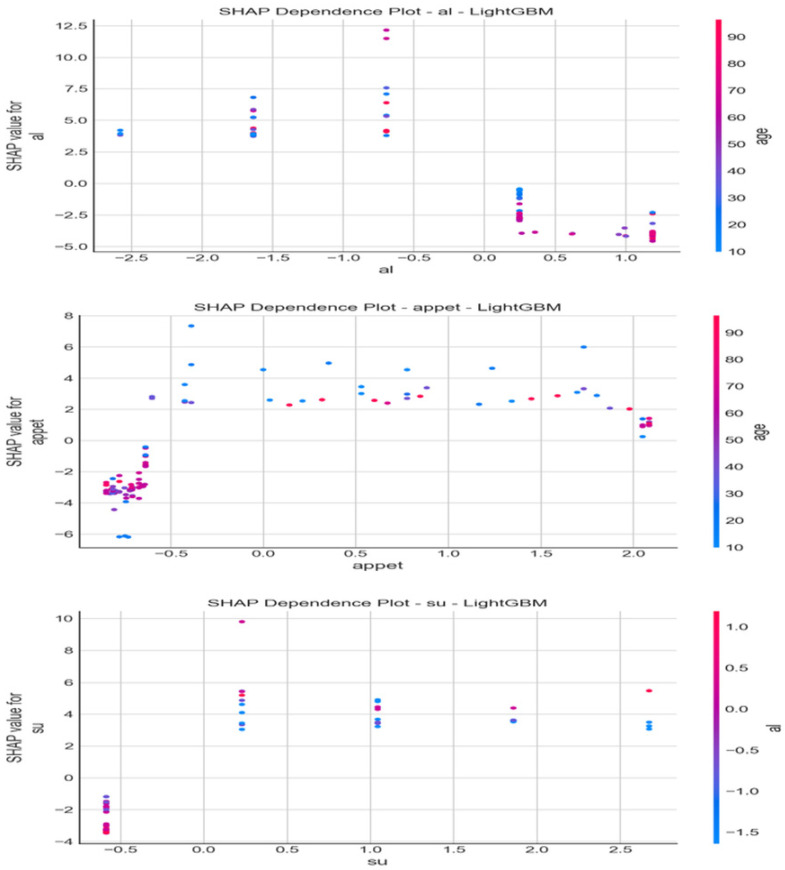
Dependence plot for al, appet, and su.

The dependence plot of al, appet, and su in Figure 8 shows that the LightGBM model does not learn linear relationships but instead learns a threshold like nonlinear relationships. Then there is a simple increase and decrease in risk. In the case of al, the regime is evident in the plot shift at which definite ranges of albumin values generate significantly greater impacts on SHAP, and the coloring emphasizes interaction by age implying that albumin-related signals can be increased within certain age groups in agreement with clinical intuition that suggests older patients have a narrower physiological margin and increased base level vulnerability. In the case of appet, the model shows a step-like shift of negative to positive SHAP contributions endorsing a clinically interpretable pattern: appetite-related status may serve as a practical syndrome-level indicator which consolidates nutrition effects of inflammation and uremia into a quantifiable signal. Considering su (sugar), the clusters are discrete owing to the categorical nature of this variable. There are significant differences in the SHAP value during the transitions between categories, indicating that the hypothesis on the ability of urine sugars to significantly change the probability of CKD is valid. Such a phenomenon is more visible against the backdrop of the urine albumin pattern.

**Figure 9:**
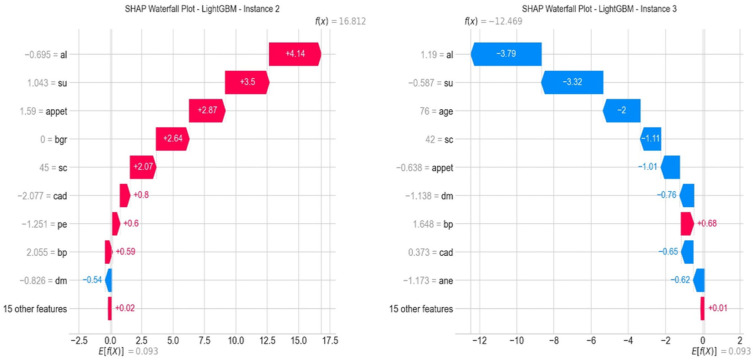
Waterfall explanations—LightGBM.

Figure 9 shows the waterfall plots. The Waterfall Instance 2 and Instance 3 on the right show how the model’s predictions can be translated into case-based clinical narratives. Example 2 (high risk): In this scenario, the predicted risk is enhanced by a prominent cluster of abnormalities, such as albumin (al), sugar (su), and appetite (appet), backed up by blood glucose (bgr) and serum creatinine (sc), with further reinforcement from comorbidity features and other clinical context indicators (e.g., blood pressure (bp), physical examination (pe), and coronary artery disease (cad)). This reflects the holistic thinking of the clinician. CKD risk is not determined by a singular value in the lab, but rather it is the confluence of trends in urine results, renal function measurements, metabolic markers, and systemic manifestations. On the contrary, in the case of low risk, Instance 3 demonstrates high negative contributions. Using the same key features (notably al and su) the combination of these factors tends to cause the prediction to shift toward non-CKD even when each factor on its own has a small negative effect of this type. It is clinically significant as it demonstrates why the model is not overreactive to a single abnormal sign: it balances the consideration of whether the overall profile of biomarkers is consistent with CKD physiology internally or not.

Overall, these SHAP views show that LightGBM is not only highly accurate on Dataset 1, but also clinically interpretable at both the population and individual levels: it prioritizes urine and renal-function markers, captures nonlinear “risk regime” behavior, and produces patient-specific explanations that resemble real diagnostic synthesis, supporting safer translation of the model into screening or decision-support workflows.

**Figure 10:**
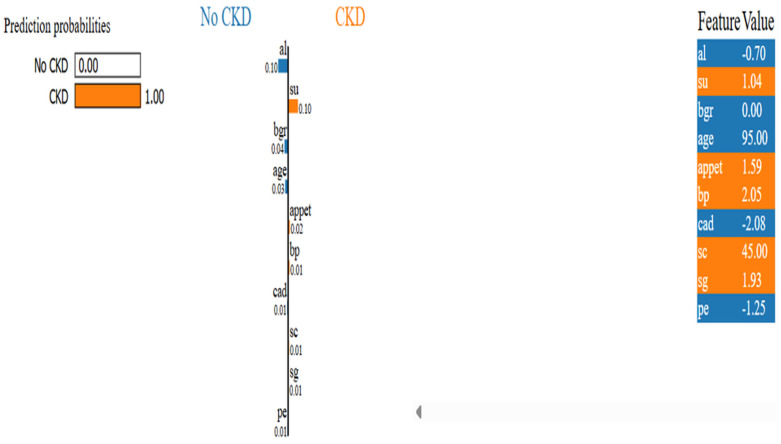
LIME Case CKD.

**Figure 11:**
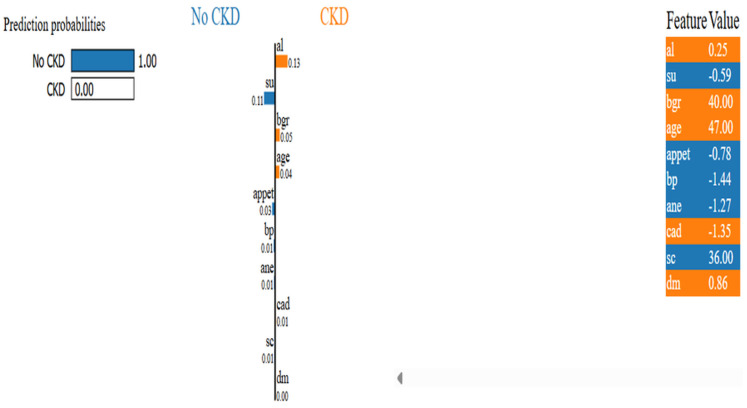
LIME Case No CKD.

To complement the global SHAP findings, local interpretable model-agnostic explanation (LIME) was used to provide case-level clinical narratives for LightGBM, illustrating why a specific patient was classified as CKD or No-CKD. Figure 10 shows LIME Case CKD and Figure 11 shows LIME Case No-CKD; the model’s predicted class probability is shown on the left, while the central bars quantify how the most influential features push the prediction toward CKD (right) or No-CKD (left), with the corresponding patient-specific feature values listed on the right. This local perspective is clinically valuable because it mirrors real practice: clinicians interpret CKD risk by integrating a small set of decisive abnormalities (urinalysis findings, filtration markers, metabolic indicators, and clinical signs), rather than relying on a single variable.

In the CKD-positive example, the model predicts a very high probability of CKD (approximately 1.00) and LIME traces this prediction to a convergent clinical pattern that was largely driven by urinalysis results and results in renal function. The most powerful motivators to CKD are urine albumin (al) and urine sugar (su) with the help of specific gravity (sg) and serum creatinine (sc)—a product that is clinically aligned with kidney damage and impaired filtration physiology. Further input of blood pressure (bp) as well as the presence of systemic and clinical responses (e.g., pedal edema (pe)) also supports the CKD interpretation, and it is not a secret that CKD is directly related with hypertension and volume overload manifestations. Critically, the explanation does not act like a strict rule (e.g., high creatinine = CKD); rather, it demonstrates that LightGBM believes most in CKD in cases when several abnormalities relevant to kidney co-exist, which can also be explained by nephrology logic and minimize the risk of overreaction to an anomalous or noisy measurement.

In the No-CKD scenario, the model gives a very confident No-CKD probability (approximately 1.00), and in LIME, the opposite scenario is displayed, where the same clinically meaningful markers have the opposite effect, and the overall effect is that the prediction is pushed out of CKD. In this case, the local decision is dominated by the lower-risk patterns in urinalysis (notably, al and su leading to No-CKD) and the more reassuring context of renal function (e.g., sc leading to No-CKD in this case) but different factors such as age and metabolic context (e.g., bgr) modify the local decision somewhat, but in minor ways. According to the clinical deployment perspective, this constitutes precisely the behavior that screening support would wish. The model is capable of being very decisive when the overall constellation of biomarkers points to no kidney disease, including the presence of any single demographic or comorbidity marker, and places an emphasis on overall physiological integrity over individual feature triggering.

Overall, the LIME descriptions show that the suggested framework can develop clinically interpretable local decisions: (i) CKD predictions are motivated by a combined signal of urinalysis abnormalities and renal filtration markers as well as supported by clinical correlates (e.g., blood pressure/edema), and (ii) No-CKD predictions are justified when these most significant biomarkers support the indication of low risk. This enhances clinical trust by demonstrating that high performance of the model is obtained by using medically plausible decision logic that can ultimately be audited at the patient level and used to describe why a patient was flagged (or not flagged) during CKD screening.

### 5.2. Dataset 2

**Figure 12:**
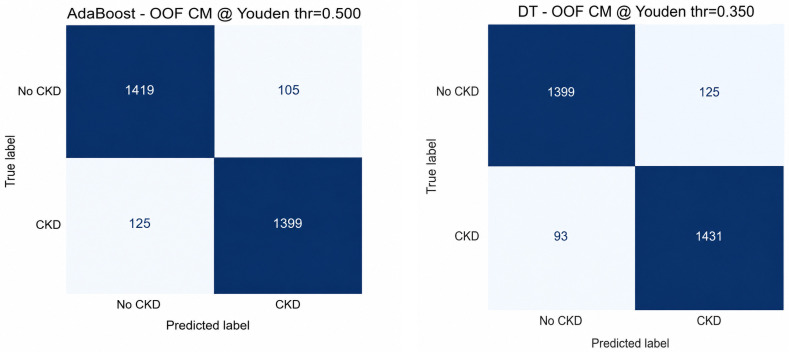

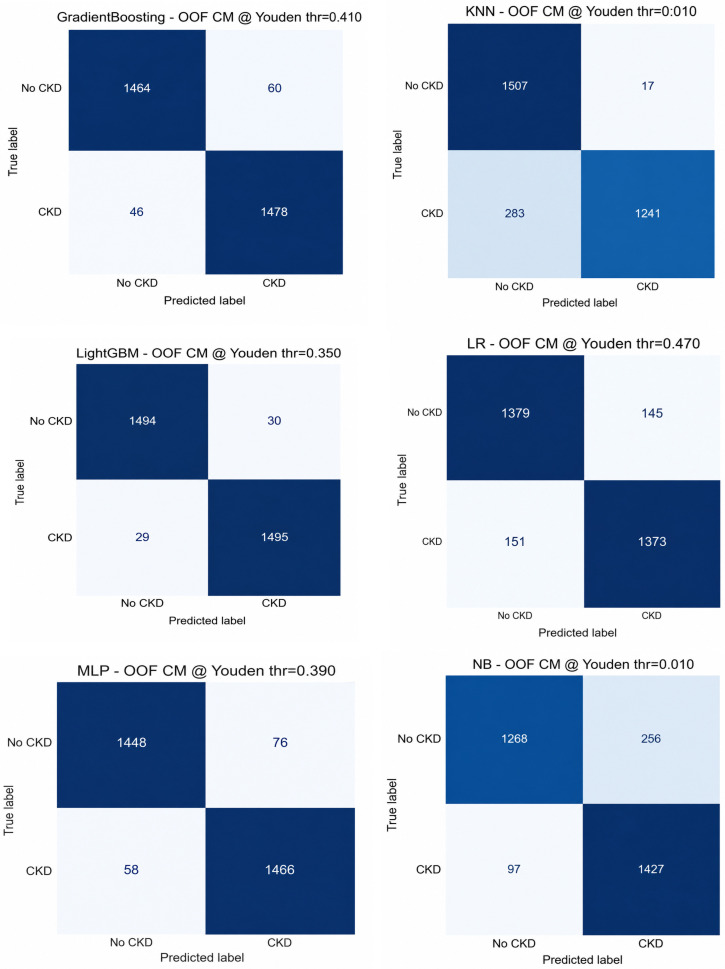

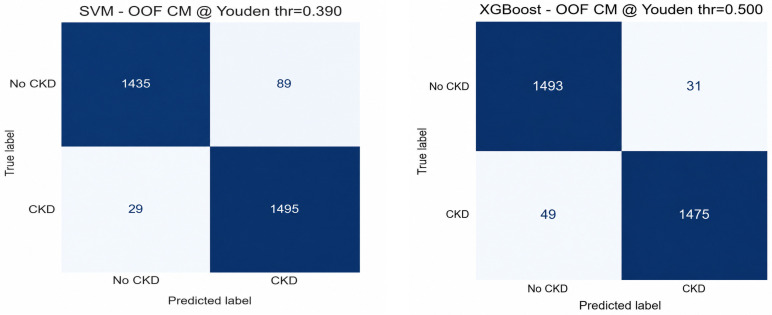
Confusion matrix of Dataset 2.

In this subsection, the outcomes of Dataset 2 will be described. Dataset 2 is larger and richer in features compared to Dataset 1 and will give a complementary analysis of the suggested framework. The analysis is concerned with the behavior of the tested classifiers on this more clinical profile as indicated by overall discrimination, operation point performance at the OOF best Youden threshold and the resulting compromise between CKD case capture and false-alarm control. Figure 12 displays the confusion matrix of each classifier operating at the chosen operating point, providing a deployment-relevant intuitive understanding of the results of the classification between CKD and non-CKD within this cohort.

**Figure 13:**
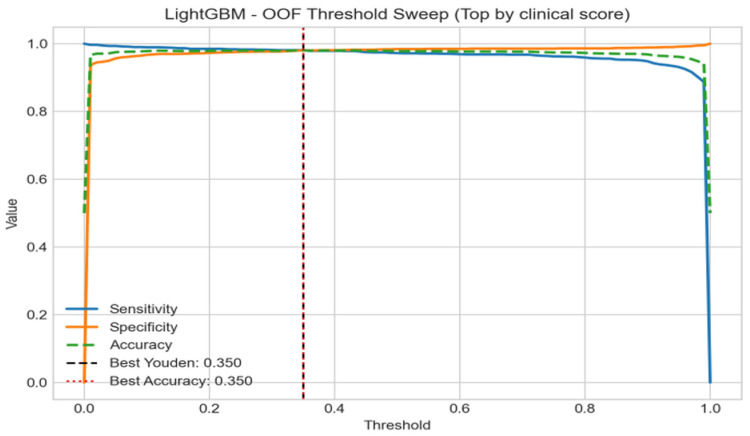
OOF threshold sweep for the top-ranked model (LightGBM).

The results obtained from Dataset 2 are shown in Table 7, which indicates that gradient boosting ensemble superiority is not cohort-dependent. In Dataset 2, LightGBM once again takes the first position with almost a ceiling of discrimination and a small range of sensitivity–specificity ratio at the optimal Youden threshold. The hierarchy of performance which is found in Dataset 1 is maintained; XGBoost and Gradient Boosting makes an apparent second tier, and classical baselines agree less and are less stable in clinical balance. It is worth noting that Dataset 2 further increases the difference between boosting algorithms and traditional classifiers, highlighting the tree-based ensemble scalability and resilience in more heterogeneous data conditions. Notably, the LightGBM OOF threshold sweep (Figure 13) has a larger plateau of equally high sensitivity and specificity around the optimal threshold (0.35) in comparison with Dataset 1. This suggests that it is more stable at the operating point, i.e., minor changes in threshold will not lead to clinically significant changes in the false-negative or false-positive rate. Concerning a screening point of view, this stability increases the trust in deployment since any reliable CKD detection should be able to withstand a small amount of calibration drift without causing harm to the patient.

In general, the fact that the cross-dataset ranking is consistent, along with better threshold stability on Dataset 2, supports the idea that LightGBM is not only a statistically most robust model, but also the solution with the most straightforward deployment. The combination of strength of discrimination, balance of errors, and strong operating in both cohorts is a great defense in its choice as the next step in the explainability and clinical interpretability analysis.

**Figure 14:**
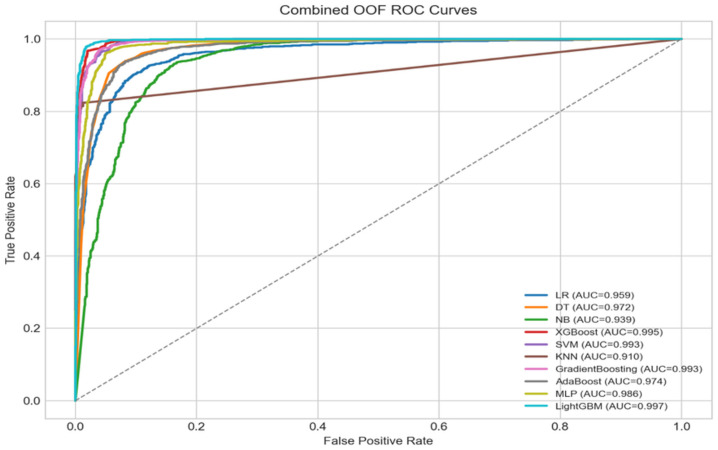
OOF ROC curves of all classifiers.

**Figure 15:**
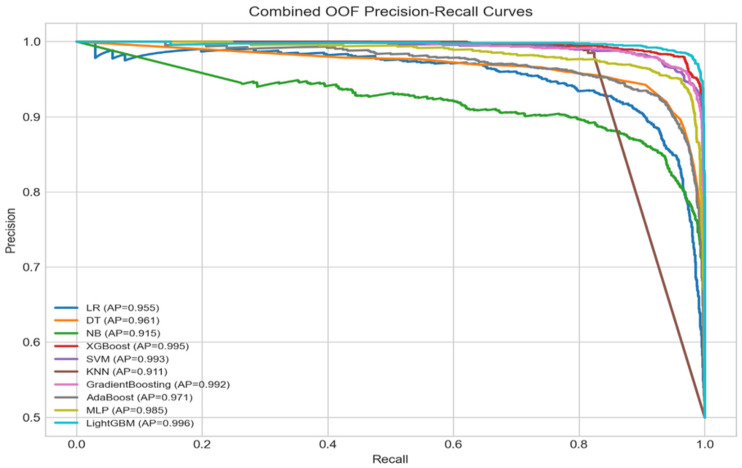
OOF precision–recall (PR) curves of all classifiers.

Figure 14 and Figure 15 give a threshold-free response that the performance ranking of Dataset 1 is maintained in Dataset 2 and responds to the behavior of the model in a larger, more heterogeneous cohort. LightGBM and XGBoost in the ROC domain (Figure 14) are near-ceiling and heavily concentrated on the upper-left part, which suggests that the outstanding separability observed in Dataset 1 applies well to Dataset 2, as opposed to being cohort-specific. Nevertheless, in contrast to Dataset 1 in which a few models were close to ideal ROC/PR behavior, the ROC curves of Dataset 2 are more spaced out between methods, especially between traditional learners (e.g., Naïve Bayes, KNN, and some linear baselines) and more complex methods, and with more feature variation in Dataset 2.

Cross-dataset contrast can also be emphasized by the precision–recall analysis as in Figure 15. Although the average precision of Dataset 1 was uniformly high in all of the models, Dataset 2 displays a more definite performance difference in precision–recall space: the boosting ensembles (LightGBM, XGBoost, and Gradient Boosting) continue to be very precise over a broad recall range, and the less powerful approaches more noticeably lose their precision as they move towards the sensitivity of a screening test. The change is clinically essential owing to the increased susceptibility of PR curves to class disproportion and false-positive load; hence, Dataset 2 is more exposed to the models that can maintain high positive predictive value and are responsible as much as possible for CKD case capture. The PR/ROC comparisons between the two datasets, combined, validate two findings: to begin with, the best boosting models are always robust across the cohorts; and finally, Dataset 2 is more effective to distinguish deployment-ready models with a greater emphasis on precision stability in the operating requirements on high recall, which further supports the argument that LightGBM is the most reliable screening-focused classifier in both tests.

**Figure 16:**
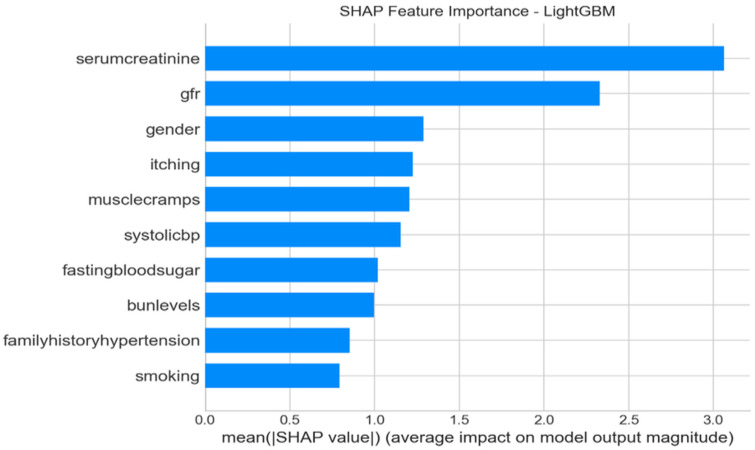
SHAP feature importance of Dataset 2 using LightGBM.

The global SHAP with important features of Dataset 2 as shown in Figure 16 indicates that the two key predictors of LightGBM are serum creatinine and GFR, which dominate the predictors by a long way compared to the other variables. Such is the phenotype of CKD expected clinically: high creatinine indicates decreased clearance and low GFR directly codes for decreased capacity to filtrate. In addition to its renal activity, the model gives significant importance to gender, uremic symptom burden (itching and muscle cramps), hemodynamic/metabolic determinants (systolic blood pressure and fasting blood sugar) and BUN levels, family history of hypertension and smoking, which are clinically sound risk factors or symptoms of CKD progression.

**Figure 17:**
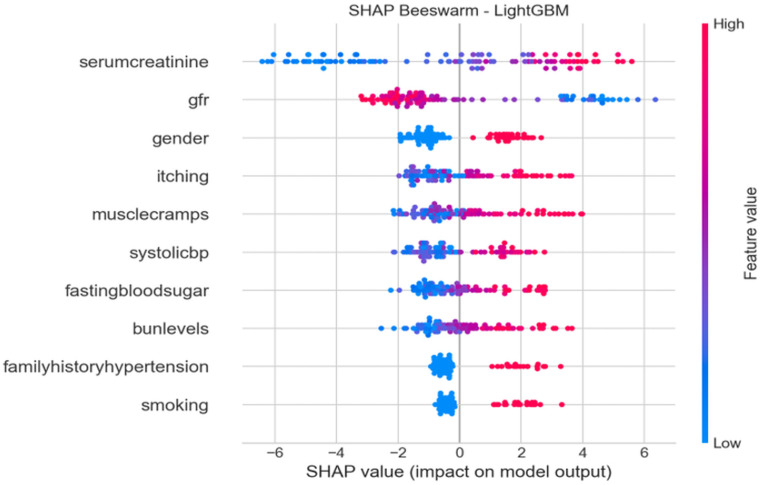
SHAP beeswarm—LightGBM.

The beeswarm plot of Dataset 2 as shown in Figure 17 explains the way these variables are driving the predictions towards CKD vs. non-CKD. The increasing values of serum creatinine always cause the SHAP values to move towards the CKD direction, and the increasing GFR moves the predictions towards non-CKD (and vice versa). Notably, the SHAP values of creatinine and GFR have broad distributions, and LightGBM does not operate under a strict rule but modifies the signal of the renal function to the situation of the patient, which is a critical feature of the screening of heterogeneous groups of individuals. An analogous clinically interpretable pattern is present in the case of supportive features: rising systolic BP, rising fasting glucose, rising BUN and rising symptom severity (itching, muscle cramps) tend to move the model to the CKD classification, consistent with established cardio-metabolic risk and uremic symptomatology behavior.

**Figure 18:**
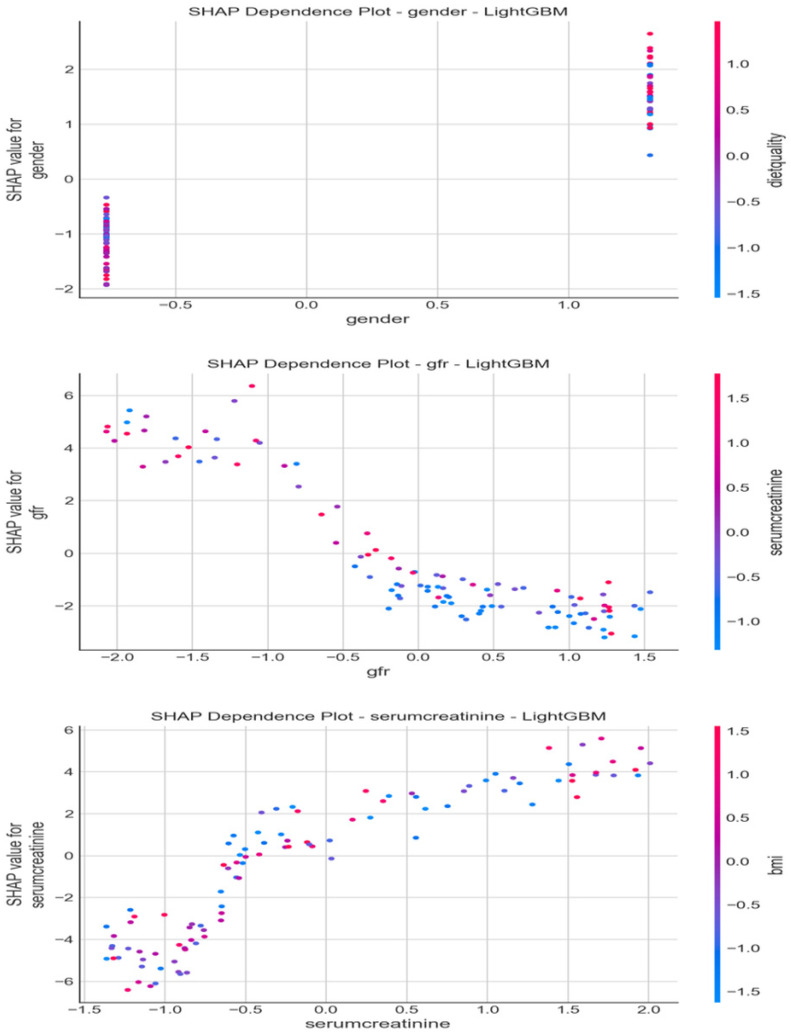
Dependence plot for gender, GFR, and serum creatinine.

The dependence plots (Figure 18, gender, GFR, and serum creatinine) indicate that the LightGBM model uses nonlinear relationships that are structured and physiologically plausible as opposed to making use of naive linear trends. In the case of serum creatinine, the dependence curve will show a strong monotonic increase in SHAP values with increases in creatinine. Strongly negative SHAP contributions (protective effect) are related to low creatinine values, and high values quickly revert the prediction to CKD. The almost-linear positive curve shows that creatinine is a prevailing biochemical severity parameter incorporated into the model. Notably, the color effect on the BMI is that the effect of the creatinine elevation could be modulated by the metabolic burden, indicating the established association existing between obesity, metabolic syndrome, and renal stress.

In the case of GFR, an inverse nonlinear relationship has been noticed. An increasing GFR gives a strongly negative SHAP contribution (protective renal functioning), whereas a decreasing GFR gives a sharply positive SHAP impact. The steeper slope of the lower GFR area is reflective of the clinical fact that disproportionate risk accrual in renal failure is found at advanced stages. Biological coupling of the interaction of coloring with serum creatinine is confirmed by the fact that patients with low GFR and high creatinine have a higher signal of CKD risk. This two-way physiological similarity of the two biomarkers strengthens mechanism validity.

In the case of gender, the model does not consider sex as a nominal input category, but it gives a directional effect systematically. One of the gender categories always allows a positive shift of the SHAP values, which suggests high modeled risk, and the other makes a negative shift. The interplay with diet quality indicates that the environmental or behavioral modifiers affect the expression of gender-specific biological predisposition in the risk of CKD. This pattern should not be seen as the manifestation of bias, but epidemiological disparities in CKD progression, comorbidity burden and healthcare behavior. Generally, the dependence plots substantiate the fact that core nephrological principles are embedded in the LightGBM model: the increasing serum creatinine and decreasing GFR are nonlinear accelerators of the estimated CKD risk, but the risk increase is stronger with increasing clinically relevant thresholds. The pattern of interaction indicates that the model is based on multivariate mechanisms of renal stress as opposed to the effect of individual features. Datasets 2 has more profound physiology as compared to Dataset 1.

**Figure 19:**
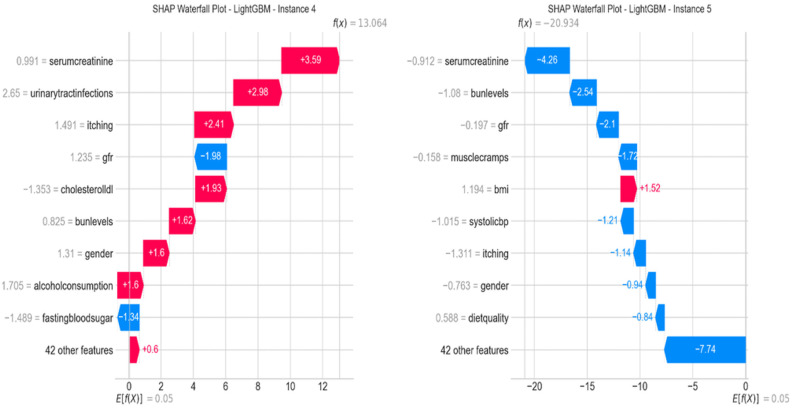
Waterfall explanations—LightGBM.

The waterfall descriptions shown in Figure 19 transform the decision process by LightGBM into case-based clinical scripts consistent with reasoning at the bedside in nephrologists. In an instance with a high likelihood of the event (Instance 4; left panel), a convergent risk signature dominated by high serum creatinine (largest positive prediction) along with urinary tract infection and itching together demonstrates a clinically plausible process of renal dysfunction with overall symptom burden. Notably, there is also multi-domain reinforcement in the model due to BUN, gender and alcohol consumption which means that the end decision is not elicited by an individual marker but is the result of the accumulation of risks across biochemical impairment, clinical symptoms and context. Interestingly, GFR will play the opposite role here and is an indication of partial physiological compensation or heterogeneity in the profile, but the cumulative effect evidence is strong enough to provide a high-risk assignment, an interpretation that is manifested in real-world practice where discordant signals can occur but do not invalidate an overall high-risk phenotype.

However, the converse scenario is the case of low risk (Instance 5; right panel), where the model shifts to non-CKD under a coherent protective profile, which is based predominantly on the lower serum creatinine, lower BUN, and more desirable GFR-related effect, and reinforced by other negative effects of systolic blood pressure, itching, gender, and diet quality. Clinically, this implies that LightGBM is very conservative with its decision-making approach when faced with conflicting data. Even though high BMI increases the probability of developing CKD, LightGBM does not overestimate this single risk factor since the other markers of the kidneys do not indicate kidney problems. This is clinically useful in the sense that it indicates evidence integration, which minimizes the possibility of using unnecessary alarms due to isolated abnormalities. Overall, the waterfall descriptions prove that the predictions of the model can be clinically synthesized: in Dataset 2, LightGBM gives more emphasis to biologically dominant renal biomarkers (serum creatinine, GFR, and BUN) and correctly incorporates symptoms and contextual modifiers, which can be characterized as individualized explanations that are similar to real diagnostic aggregation instead of black-box choices. In contrast to the interpretations for Dataset 1, which relied extensively on parameters related to urine as well as categorically coded variables, those for Dataset 2 are based on biochemical interpretations and stage-related analysis. As such, Dataset 2 is better suited for use as a screening or decision tool.

**Figure 20:**
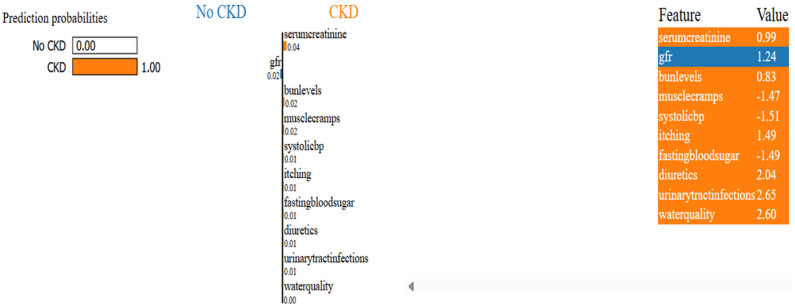
LIME Case CKD.

**Figure 21:**
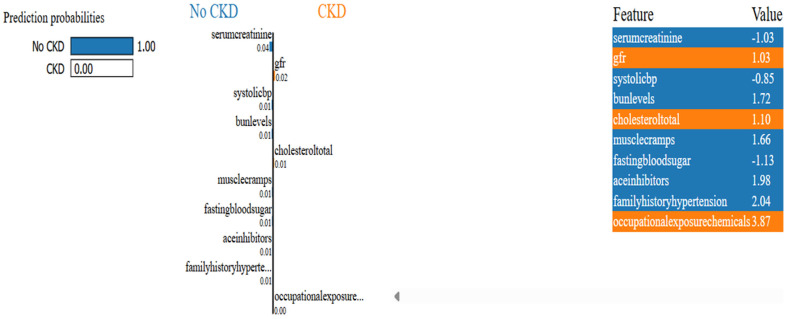
LIME Case No-CKD.

To supplement the global SHAP results of Dataset 2, LIME was implemented to produce clinical narratives of patients of the highest-ranked LightGBM model, explaining why one or another person is identified as CKD or Non-CKD. Figure 20 (CKD case) and Figure 21 (No-CKD case) illustrate the predicted class probability on the left, and the central contribution bars indicate how the most significant variables move the decision to CKD (right) or the No-CKD (left), and the patient-specific feature values are listed on the right. For CKD, the model is based on an informative pattern that includes features of renal dysfunction along with symptom and contextual features. This includes mainly serum creatinine, which is confirmed by GFR, BUN, and symptom/management features like itching, muscle cramps, and use of diuretics as well as contextual features like water quality, UTI, etc. The outcome produced by such an algorithm is therefore realistic considering what can be expected from such dysfunction. By contrast, in No-CKD the model is pulled unambiguously away from CKD by a more internally consistent protective pattern of the renal markers (serum creatinine and GFR) even with individual contextual risk factors (e.g., cholesterol, occupational exposure to chemicals, or family-history factors) having a small contribution. This local interpretability is critically important clinically since it captures the way real screening choices are determined, where a small combination of determinative abnormalities leads to case-by-case justification across all filtration markers, all metabolic profiles, all symptoms, all treatment cues and exposures, and thus is justifiable in case-by-case descriptions of all decisions available to CKD decision-support practices.

The proposed CKD detection framework has shown consistent and clinically relevant performance on both datasets. In addition, the results indicated that population-specific characteristics may influence the relative importance and diagnostic value of clinical biomarkers, stressing the need for context-aware interpretation of machine learning predictions in CKD screening and diagnosis. In Dataset 1, gradient boosting models—most notably LightGBM—demonstrated near-ceiling discriminatory performance with a highly stable Youden-optimized operating point. This configuration provided an effective balance between minimizing missed CKD cases and reducing false alarms. Furthermore, the explanatory features consistently converged on canonical nephrology cues, including urinalysis markers and renal-function surrogates such as albumin/glucose profiles and creatinine-associated physiology. Dataset 2 adds a more sophisticated feature space that resembles the real world, including symptoms, medications, behaviors, and exposures. Risk is signaled more directly in Dataset 2. The interpretability framework introduces a clinically robust dimension: serum creatinine and GFR remain the main anchors, but risk is also contextually shaped by symptom burden (e.g., pruritus, muscle cramps), treatment settings, and environmental or occupational indicators. This reflects the multifactorial interaction that clinicians consider when stratifying CKD remotely, beyond purely laboratory-based assessments. All of these findings are consistent: the framework is not merely accurate but threshold-sensitive and clinically interpretable even in heterogeneous data regimes, and can be deployed with a high level of calibration so that a decision threshold can be adjusted in a way that balances local screening priorities and explanations that are consistent with nephrology logic at both population and individual levels contributing to enhanced trust, auditability and real-world translation of CKD decision support.

## 6. Conclusions and Future Directions

In this study, we propose a clinically meaningful and interpretable machine learning framework for detection of chronic kidney disease (CKD). The proposed approach was rigorously evaluated on two heterogeneous public datasets using a stratified 10-fold cross-validation protocol with out-of-fold (OOF) predictions to ensure robust and unbiased performance estimation. The framework integrated predictive modeling and explainability techniques to offer robust diagnostic performance and transparent, biomarker-driven insights for clinical interpretation and decision-making. In both cohorts, gradient boosting models always provided the highest discrimination, as well as the most predictive threshold-based screening behavior, and LightGBM became the best-performing classifier, both in terms of strong ROC-AUC/PR-AUC performance and a clinically sensible operating point, identified using the Youden criterion. In addition to its accuracy, the framework was developed with clinical interpretability as a core objective. SHAP analyses were structured to yield both global and local explanations that align with nephrological reasoning—whereby renal-function markers such as serum creatinine and GFR are given a priori precedence and were presented in formats amenable to interpretation within clinical symptoms and lifestyle contexts, as demonstrated through LIME case narratives. All of these results suggest that the proposed pipeline could be used to facilitate feasible CKD screening and risk stratification, as it provides high levels of generalization across datasets, clear management of the sensitivity–specificity tradeoff during deployment, and explainable mechanisms, which would facilitate trust and clinical auditability. Future work will aim to strengthen external validity and ensure that the models are clinically prepared for deployment in real-world settings.

To determine the transportability of the framework across populations, measurement protocols, and prevalence changes, future work will first involve checking the framework on multi-center cohorts and prospectively collected data. Second, we will aim to generalize threshold choice to cost-sensitive and utility-based criteria explicitly modeling clinical harm (false-negative risk) and resource utilization (false-positive follow-up), and we will also include more detailed calibration studies to provide sound probability results. Third, to identify trends in CKD progression and enable early intervention, we will incorporate temporal and longitudinal modeling based on repeated laboratory values and comorbidity trajectories. Fourth, explainability layer will be extended to contain clinician-facing summaries and counterfactual suggestions to aid actionable decision-making, as well as fairness audits by demographic subgroups to limit possible prejudice. Lastly, to ensure that the outputs, thresholds, and explanations provided by the model align with the actual diagnostic and care processes, we will explore lightweight deployment and real-time implementation strategies into clinical workflows (e.g., EHR-based decision support), supported by usability studies with clinicians.

This study has a limitation. Although the results were encouraging in two independent public CKD datasets, they were not validated in external clinical data in hospitals or medical laboratories. The developed framework will be tested on multi-center clinical cohorts from different healthcare settings in the future to further evaluate its generalizability, robustness and clinical utility across patient populations and laboratory settings. Furthermore, prospective validation studies and integration into clinical decision-support systems will be discussed to help safe and reliable deployment in clinical nephrology practice. Another important direction for future research is the systematic evaluation of class imbalance management techniques. Even though SMOTE was used in the current framework to mitigate class imbalance while training, doing a more dedicated ablation that compares results with vs. without oversampling ended up being outside the scope here. Future work will investigate how different resampling approaches such as SMOTE, Borderline-SMOTE, ADASYN, and cost-sensitive learning shape predictive performance calibration, threshold stability and model interpretability across a variety of CKD cohorts.

## Figures and Tables

**Table 1:** Related work comparison.

Study	Task	Dataset	Models/Method	Validation	Best Reported Results	XAI	Threshold Strategy Reported?	Key Limitations/Notes
[31]	DKD prediction (classification)	410 samples, 18 attributes (WEKA)	9 ML models (IBK/KNN, Random Tree, RF, J48, NB, MLP, AdaBoostM1, etc.)	10-fold CV	Best: 93.6585% accuracy (IBK/Random Tree), kappa 0.8731	No	No (primarily accuracy/error metrics)	Single dataset; no SHAP/LIME; limited clinical operating-point reporting
[9]	CKD progression/kidney failure prediction	Pathology-based cohorts (Australia + Japan)	DT/RF with eGFR-derived longitudinal features; imbalance under-sampling; transfer learning	Internal + external validation; fine-tuning on 15% target cohort	AUC: 0.94–0.98 (internal), 0.88–0.93 (external)	SHAP + LIME (+ counterfactuals)	Not explicit (focus on ROC-AUC)	Targets progression (not binary detection); relies on longitudinal eGFR dynamics
[32]	Binary CKD prediction	UCI CKD (400, 25 features)	MLP + MI feature selection (Top-12); baselines: Ridge, SGD, BNB, LR, GNB, RF, DT	75/25 hold-out split	Reported 100% metrics for MLP	LIME	No (default classification; no clinical thresholding)	Single dataset; single split (no OOF); potential optimistic bias; no SHAP
[33]	CKD severity grouping (stages 1–2 vs. 3–5)	Clinical dataset (CKD staging groups)	XGBoost vs. LR/RF/DT/NB; hyperparameter search	(As reported in study)	XGBoost best: Acc 93.29%, AUC 0.9689	SHAP + LIME	Not explicit (reports Acc/Sens/Spec; threshold method not detailed)	Stage grouping task; operating-point selection not standardized to OOF-Youden style
[34]	CKD stage classification	Single hospital cohort (India)	DL + feature selection via MHMXAI (ESA + GSR-TEO + SHAP/LIME)	Multiple comparisons + statistical tests (Friedman/Nemenyi)	CNN best; ~98–99.5% accuracy (reported range)	SHAP + LIME (within FS strategy)	No (primarily accuracy-driven staging)	Single site; staging focus; external generalization uncertain
[35]	Binary CKD detection (screening)	Single-center BMC cohort	Multiple ML + ensembles (Voting/Stacking)	5-fold & 10-fold stratified CV	RF best ~90–92% accuracy (reported across CV schemes)	Not SHAP/LIME-focused	No (no Youden/clinical threshold described)	Single center; cross-sectional; limited sociodemographic richness
[36]	Mortality prediction in CKD	KNHDIS (Korea), 12,680 CKD; temporal split 2016–2020 vs. 2021	DL model (MLP) + LASSO feature selection; baselines RF/XGBoost	Temporal hold-out test (2021)	AUC 0.83, accuracy ~96.8%	No SHAP/LIME	No (focus on AUC/accuracy)	Prognosis (mortality), not CKD detection; no biomarker-driven modeling; limited interpretability
[37]	CKD risk stratification	Clinical CKD dataset (as reported)	Optuna (TPE) tuning + XGBoost + SHAP	(As reported in study)	XGBoost: Acc 92.4%, AUC 97.7%	SHAP	Not explicit (thresholding not emphasized)	Single region/cross-sectional; calls for broader validation
Our work	Binary CKD prediction (CKD vs. non-CKD)	Two public Kaggle datasets	10 ML models: LightGBM, XGBoost, Gradient Boosting, AdaBoost, DT, LR, NB, MLP, KNN, SVM; unified pipeline + SHAP/LIME	Stratified 10-fold CV + OOF predictions	Report OOF ROC-AUC/PR-AUC/Brier + OOF @Youden: Sens, Spec, MCC, Acc, F1; also CV mean ± std + runtime	SHAP + LIME	Yes—Youden’s J (OOF)	Strength: consistent evaluation across 2 datasets + clinically oriented operating-point reporting + dual explainability; limitation: public data (future external clinical cohort validation)

**Table 2:** Dataset 1 list of features.

Feature ID	Variable Name	Type	Description	Units
F1	age	Integer	—	year
F2	bp	Integer	Blood pressure	mm/Hg
F3	sg	Categorical	Specific gravity	—
F4	al	Categorical	Albumin	—
F5	su	Categorical	Sugar	—
F6	rbc	Binary	Red blood cells	—
F7	pc	Binary	Pus cell	—
F8	pcc	Binary	Pus cell clumps	—
F9	ba	Binary	Bacteria	—
F10	bgr	Integer	Blood glucose random	mgs/dL
F11	bu	Integer	Blood urea	mgs/dL
F12	sc	Continuous	Serum creatinine	mgs/dL
F13	sod	Integer	Sodium	mEq/L
F14	pot	Continuous	Potassium	mEq/L
F15	hemo	Continuous	Hemoglobin	gms
F16	pcv	Integer	Packed cell volume	—
F17	wbcc	Integer	White blood cell count	cells/cmm
F18	rbcc	Continuous	Red blood cell count	millions/cmm
F19	htn	Binary	Hypertension	—
F20	dm	Binary	Diabetes mellitus	—
F21	cad	Binary	Coronary artery disease	—
F22	appet	Binary	Appetite	—
F23	pe	Binary	Pedal edema	—
F24	ane	Binary	Anemia	—
	class	Binary	CKD or not CKD	—

**Table 3:** Dataset 2 list of features.

Feature ID	Variable Name	Type	Description	Units
F1	Age	Integer	Patient age (20–90)	years
F2	Gender	Binary	0 = Male, 1 = Female	—
F3	Ethnicity	Categorical	0 = Caucasian, 1 = African American, 2 = Asian, 3 = Other	—
F4	Socioeconomic Status	Categorical	0 = Low, 1 = Middle, 2 = High	—
F5	Education Level	Categorical	0 = None, 1 = High School, 2 = Bachelor’s, 3 = Higher	—
F6	BMI	Continuous	Body mass index (15–40)	kg/m^2^
F7	Smoking	Binary	0 = No, 1 = Yes	—
F8	Alcohol Consumption	Continuous	Weekly alcohol intake (0–20)	units/week
F9	Physical Activity	Continuous	Weekly physical activity (0–10)	hours/week
F10	Diet Quality	Continuous	Diet quality score (0–10)	score
F11	Sleep Quality	Continuous	Sleep quality score (4–10)	score
F12	Family History Kidney Disease	Binary	Family history of kidney disease (0/1)	—
F13	Family History Hypertension	Binary	Family history of hypertension (0/1)	—
F14	Family History Diabetes	Binary	Family history of diabetes (0/1)	—
F15	Previous Acute Kidney Injury	Binary	Prior acute kidney injury (0/1)	—
F16	Urinary Tract Infections	Binary	History of urinary tract infections (0/1)	—
F17	Systolic BP	Integer	Systolic blood pressure (90–180)	mmHg
F18	Diastolic BP	Integer	Diastolic blood pressure (60–120)	mmHg
F19	Fasting Blood Sugar	Continuous	Fasting blood sugar (70–200)	mg/dL
F20	HbA1c	Continuous	HbA1c (4.0–10.0)	%
F21	Serum Creatinine	Continuous	Serum creatinine (0.5–5.0)	mg/dL
F22	BUN Levels	Continuous	Blood urea nitrogen (5–50)	mg/dL
F23	GFR	Continuous	Glomerular filtration rate (15–120)	mL/min/1.73 m^2^
F24	Protein in Urine	Continuous	Urine protein (0–5)	g/day
F25	ACR	Continuous	Albumin-to-creatinine ratio (0–300)	mg/g
F26	Serum Electrolytes Sodium	Continuous	Serum sodium (135–145)	mEq/L
F27	Serum Electrolytes Potassium	Continuous	Serum potassium (3.5–5.5)	mEq/L
F28	Serum Electrolytes Calcium	Continuous	Serum calcium (8.5–10.5)	mg/dL
F29	Serum Electrolytes Phosphorus	Continuous	Serum phosphorus (2.5–4.5)	mg/dL
F30	Hemoglobin Levels	Continuous	Hemoglobin (10–18)	g/dL
F31	Cholesterol Total	Continuous	Total cholesterol (150–300)	mg/dL
F32	Cholesterol LDL	Continuous	LDL cholesterol (50–200)	mg/dL
F33	Cholesterol HDL	Continuous	HDL cholesterol (20–100)	mg/dL
F34	Cholesterol Triglycerides	Continuous	Triglycerides (50–400)	mg/dL
F35	ACE Inhibitors	Binary	ACE inhibitor use (0/1)	—
F36	Diuretics	Binary	Diuretic use (0/1)	—
F37	NSAID Use	Integer	NSAID use frequency (0–10)	times/week
F38	Statins	Binary	Statin use (0/1)	—
F39	Antidiabetic Medications	Binary	Antidiabetic medication use (0/1)	—
F40	Edema	Binary	Edema present (0/1)	—
F41	Fatigue Levels	Continuous	Fatigue severity (0–10)	score
F42	Nausea Vomiting	Integer	Nausea/vomiting frequency (0–7)	times/week
F43	Muscle Cramps	Integer	Muscle cramps frequency (0–7)	times/week
F44	Itching	Continuous	Itching severity (0–10)	score
F45	Quality of Life Score	Continuous	Quality of life (0–100)	score
F46	Heavy Metals Exposure	Binary	Heavy metals exposure (0/1)	—
F47	Occupational Exposure Chemicals	Binary	Occupational chemical exposure (0/1)	—
F48	Water Quality	Binary	0 = Good, 1 = Poor	—
F49	Medical Checkup Frequency	Integer	Medical checkups per year (0–4)	visits/year
F50	Medication Adherence	Continuous	Adherence score (0–10)	score
F51	Health Literacy	Continuous	Health literacy score (0–10)	score
	Diagnosis	Binary	CKD diagnosis status (0 = No, 1 = Yes)	—

**Table 4:** Experimental parameter settings.

Component	Setting
Random seed	RANDOM_STATE = 42
Validation protocol	Stratified 10-fold CV (repeated in the implemented protocol)
Number of Run	20
Numeric imputation	KNN_NEIGHBORS = 5
Categorical imputation	Most-frequent strategy
Scaling	Standardization (z-score scaling)
Outlier screening	z-score threshold = 3.0
Imbalance handling	Dataset balancing via SMOTE
Threshold selection	OOF-based Youden’s J
SHAP settings	SHAP_SAMPLE_N = 100, SHAP_BACKGROUND_SIZE = 50
LIME settings	LIME_N_FEATURES = 10

**Table 5:** Hyperparameter settings of the classifiers.

Model	Hyperparameters Setting
Logistic Regression (LR)	solver = liblinear; max_iter = 2000; class_weight = balanced; random_state = 42
Decision Tree (DT)	criterion = gini; max_depth = None; class_weight = balanced; random_state = 42
Gaussian Naïve Bayes (NB)	GaussianNB (default); var_smoothing = 1 × 10^−9^
SVM (RBF)	kernel = rbf; C = 1.0; gamma = scale; probability = True; class_weight = balanced; random_state = 42
KNN	n_neighbors = 7; weights = distance; metric = minkowski
MLP	hidden_layer_sizes = (128, 64); activation = relu; solver = adam; max_iter = 1500; early_stopping = True; random_state = 42
AdaBoost	n_estimators = 400; learning_rate = 1.0; random_state = 42
Gradient Boosting	n_estimators = 400; learning_rate = 0.1; max_depth = 3; random_state = 42
XGBoost	booster = gbtree; n_estimators = 600; max_depth = 4; learning_rate = 0.03; subsample = 0.9; colsample_bytree = 0.9; min_child_weight = 1.0; reg_lambda = 1.0; eval_metric = logloss; n_jobs = −1; random_state = 42 (scale_pos_weight set per fold)
LightGBM	boosting = gbdt; n_estimators = 600; num_leaves = 31; max_depth = −1; class_weight = balanced; n_jobs = −1; verbose = −1; random_state = 42

**Table 6:** Results obtained from Dataset 1.

Rank	ML Model	Clinical Composite Score	OOF best Youden Thr	OOF Youden Jmax	OOF Roc_Auc	OOF Recall @Youden	OOF Specificity @Youden	OOF Mcc	OOF Accuracy	OOF F1	OOF Pr_Auc	OOF Brier	CV AUC	CV Sens	CV Spec	CV MCC	Train Time
1	**LightGBM**	**99.6**	0.41	**98.8**	**99.98**	99.2	**99.6**	98.8	**99.4**	**99.4**	**99.98**	0.006	**99.99 ± 0.04**	99.10 ± 1.81	99.46 ± 1.42	**98.59 ± 2.19**	0.078 ± 0.019
2	XGBoost	**99.6**	0.7	**98.8**	99.97	99.2	**99.6**	98.8	**99.4**	**99.4**	99.97	0.0071	99.98 ± 0.08	**99.16 ± 1.72**	99.04 ± 2.05	98.24 ± 2.50	0.140 ± 0.024
3	GradientBoosting	**99.6**	0.05	**98.8**	99.97	**100**	98.8	**98.81**	**99.4**	**99.4**	99.97	**0.0058**	99.99 ± 0.05	99.08 ± 1.86	99.36 ± 1.62	98.47 ± 2.34	0.691 ± 0.034
4	AdaBoost	99.47	0.48	98.4	**99.98**	99.2	99.2	98.4	99.2	99.2	**99.98**	0.1201	99.96 ± 0.11	98.06 ± 2.68	**99.66 ± 1.25**	97.78 ± 2.73	0.688 ± 0.032
5	DT	98.29	0.41	95.6	99.19	98.8	96.8	95.62	97.8	97.82	98.96	0.0209	96.94 ± 2.66	97.30 ± 3.27	96.58 ± 3.84	93.98 ± 5.25	0.003 ± 0.000
6	LR	96.91	0.57	91.6	98.93	93.2	98.4	91.72	95.8	95.69	99.12	0.0361	98.91 ± 1.26	94.22 ± 4.78	96.32 ± 3.63	90.71 ± 6.10	0.002 ± 0.001
7	NB	95.58	0.01	86.4	99.82	86.4	**100**	87.21	93.2	92.7	99.83	0.0846	99.84 ± 0.31	81.96 ± 7.73	**100.00 ± 0.00**	83.49 ± 6.61	0.001 ± 0.000
8	MLP	94.47	0.39	87.2	96.08	95.2	92	87.24	93.6	93.7	95.44	0.0726	95.46 ± 3.00	89.92 ± 6.92	91.58 ± 6.65	81.93 ± 8.27	0.086 ± 0.023
9	KNN	94.41	0.34	87.2	95.91	92	95.2	87.24	93.6	93.5	95.99	0.0623	95.58 ± 2.79	89.10 ± 6.17	95.52 ± 4.11	85.05 ± 6.61	0.001 ± 0.000
10	SVM	94.2	0.39	87.2	95.33	93.6	93.6	87.2	93.6	93.6	94.73	0.0646	95.40 ± 2.84	92.54 ± 5.13	94.08 ± 4.45	86.87 ± 5.90	0.023 ± 0.002

**Table 7:** Results obtained from Dataset 2.

Rank	ML Model	Clinical Composite Score	OOF best Youden Thr	OOF Youden Jmax	OOF Roc_Auc	OOF Recall @Youden	OOF Specificity @Youden	OOF Mcc	OOF Accuracy	OOF F1	OOF Pr_Auc	OOF Brier	CV AUC	CV Sens	CV Spec	CV MCC	Train Time
1	LightGBM	**98.64**	0.35	96.13	99.72	98.10	98.03	96.13	98.06	98.06	99.64	1.73	**99.69 ± 0.25**	**97.25 ± 1.25**	**98.11 ± 1.02**	**95.37 ± 1.56**	0.742 ± 0.144
2	XGBoost	98.13	0.50	94.75	99.53	96.78	97.97	94.76	97.38	97.36	99.48	2.53	99.49 ± 0.30	96.09 ± 1.63	97.61 ± 1.09	93.73 ± 1.89	0.859 ± 0.134
3	Gradient Boosting	97.49	0.41	93.04	99.28	96.98	96.06	93.05	96.52	96.54	99.22	2.99	99.19 ± 0.37	95.17 ± 1.70	96.47 ± 1.30	91.66 ± 2.15	15.051 ± 3.682
4	SVM	97.25	0.39	92.26	99.33	98.10	94.16	92.33	96.13	96.20	99.28	2.86	99.31 ± 0.33	97.13 ± 1.45	94.72 ± 1.79	91.89 ± 2.24	0.784 ± 0.030
5	MLP	96.65	0.39	91.21	98.60	96.19	95.01	91.21	95.60	95.63	98.47	3.96	98.49 ± 0.73	94.10 ± 2.18	95.19 ± 1.81	89.33 ± 3.01	0.564 ± 0.161
6	Decision Tree	94.39	0.35	85.70	97.25	93.90	91.80	85.71	92.85	92.92	96.13	6.18	87.49 ± 1.96	83.92 ± 3.13	91.06 ± 2.46	75.23 ± 3.88	0.155 ± 0.013
7	AdaBoost	94.17	0.50	84.91	97.37	91.80	93.11	84.92	92.45	92.40	97.13	21.44	97.15 ± 0.74	90.91 ± 2.27	92.73 ± 1.84	83.70 ± 2.82	5.313 ± 0.151
8	Logistic Regression	92.25	0.47	80.58	95.88	90.09	90.49	80.58	90.29	90.27	95.53	7.54	95.86 ± 0.96	89.13 ± 2.57	90.95 ± 2.06	80.14 ± 3.24	0.010 ± 0.002
9	KNN	90.54	0.01	80.31	91.00	81.43	**98.88**	81.57	90.16	89.22	91.13	25.51	88.52 ± 1.79	29.64 ± 3.75	**100.00 ± 0.00**	41.66 ± 3.11	0.001 ± 0.000
10	Naïve Bayes	90.37	0.01	76.84	93.90	93.64	83.20	77.26	88.42	88.99	91.47	12.11	93.88 ± 1.32	83.76 ± 2.97	89.18 ± 2.38	73.10 ± 3.76	0.003 ± 0.000

## Data Availability

The datasets supporting the findings of this study is publicly available through the following URL: Dataset 1: https://archive.ics.uci.edu/dataset/336/chronic+kidney+disease (accessed on 20 January 2026); Dataset 2: https://www.kaggle.com/datasets/rabieelkharoua/chronic-kidney-disease-dataset-analysis (accessed on 20 January 2026).
